# Ventilation‐Weaning Protocols in Children Admitted to Pediatric Intensive Care Units: Systematic Review of Randomized Trials

**DOI:** 10.1002/ppul.71436

**Published:** 2025-12-22

**Authors:** Suzana C. Almeida, Rayany C. de Souza, Ingrid G. Azevedo, Ivanízia S. da Silva, Vivian Mara G. O. Azevedo

**Affiliations:** ^1^ Graduate Program in Physiotherapy, Physical Education and Physiotherapy School Universidade Federal de Uberlândia Uberlândia Brazil; ^2^ Graduate Program in Health Sciences, Medicine School Universidade Federal de Uberlândia Uberlândia Brazil; ^3^ Universidad Católica de Temuco Temuco Chile; ^4^ Maternidade Escola Januário Cicco Natal Brazil; ^5^ Physical Education and Physiotherapy School Universidade Federal de Uberlândia (UFU) Uberlândia Brazil

**Keywords:** airway extubation, clinical protocols, intensive care units, pediatric, randomized controlled trial, ventilator weaning

## Abstract

**Objective:**

To determine whether implementing a ventilator weaning protocol in a Pediatric Intensive Care Unit (PICU) can decrease the duration of invasive mechanical ventilation (IMV), the PICU and hospital length of stay (LOS), mortality rates, and extubation failure compared to usual care.

**Methods:**

PubMed, EMBASE, CINAHL, Web of Science, and Cochrane Central Register of Controlled Trials were searched. The randomized controlled trials reporting a weaning protocol compared with usual care in a PICU were selected. The risk of bias and the certainty of evidence were assessed. The duration of IMV was the main outcome, and PICU and hospital LOS, extubation failure, and mortality were the secondary outcomes.

**Results:**

Seven trials were included (*n* = 11,742). The implemented protocols included a weaning sedation plus ventilation protocol, a weaning protocol, and automated computer‐driven weaning. The IMV time was shorter with the weaning protocol (MD −1.2, 95% CI −1.27 to −1.13; 1 trial, *N* = 260; moderate‐certainty evidence) and the automated computer drive weaning protocol (MD −2.33, 95% CI −3.42 to −1.24; 1 trial, *N* = 2199; moderate‐certainty evidence). The weaning sedation plus ventilation protocol reduced hospital LOS in children intubated for respiratory disease (MD −1.2, 95% CI −1.27 to −1.13; 1 trial, *N* = 260; moderate‐certainty evidence). However, no significant differences were observed between protocols and usual care in PICU LOS, extubation failure, or mortality.

**Conclusion:**

Limited evidence indicates that weaning protocols may shorten IMV duration and that sedation and weaning protocols may reduce hospital stay in children with respiratory disease admitted to the PICU. However, their safety and effectiveness in reducing extubation failure or mortality remain unproven. This underscores the need for high‐quality studies, as current practice is based on low‐certainty evidence.

## 1 Introduction

While invasive mechanical ventilation (IMV) is essential for supporting the lives of children in Pediatric Intensive Care Units (PICU), it can also result in complications such as ventilator‐induced lung injury, ventilator‐associated pneumonia [1, 2, 3], pneumothorax, oxygen toxicity, atelectasis, hemodynamic disturbances, reduced functional capacity, muscle weakness, and prolonged hospital stays [2, 3, 4]. Therefore, IMV should be discontinued as soon as the patient's condition allows [3, 4, 5].

Weaning from IMV is a complex process influenced by several key elements, including the patient's clinical status, the weaning method, organizational dynamics, the healthcare team's composition, and the expertise, training, and experience of the involved professionals [6, 7, 8]. The management of intubated children in the PICU is more complex than in adults due to distinct airway and pulmonary anatomy [9, 10]. Children face a higher risk of complications [11], and younger age is a recognized risk factor for extubation failure [12]. Moreover, their wide range of physiological profiles and diverse underlying medical and surgical conditions, together with the heterogeneity of pediatric respiratory diseases, make predicting and managing respiratory failure particularly challenging [13, 14]. Due to this complexity, variations in practice may arise. These can negatively affect the patient, raise the risk of extubation failure [15, 16], and, as a result, prolong the duration of IMV and increase morbidity [17, 18].

These challenges are further compounded by the limited availability of robust data to guide ventilatory strategies in this population [19], underscoring the need for tailored, evidence‐informed management approaches. A structured protocol can reduce variations in clinical practice, prevent delays, and avoid adverse outcomes from differing opinions [20, 21].

In the pediatric population, there is no consensus on the optimal methods or timing for initiating weaning and extubation [19, 22, 23]. Therefore, this systematic review aims to determine whether implementing a weaning protocol in a PICU, compared to usual care, can decrease the duration of IMV, the length of stay (LOS) in the PICU or hospital, extubation failure, and mortality rates.

## 2 Methods

This systematic review followed a prepublished protocol (International Prospective Register of Systematic Reviews Database, CRD42023399650) and adhered to Cochrane's methodology [24]. The reporting followed the Preferred Reporting Items Systematic Reviews and Meta‐Analysis (PRISMA) guidelines [25] (PRISMA 2020 Checklist).

### 2.1 Search Strategy

The PubMed, EMBASE, CINAHL, Web of Science, and the Cochrane Central Register of Controlled Trials databases were searched using combinations of synonyms for “Pediatric Intensive Care Units,” “ventilator weaning,” “clinical protocols,” and “randomized controlled trial” (Table S1). Ongoing and unpublished studies were searched in the International Clinical Trials Registration Platform, Clinicaltrials.gov, and ProQuest for theses. Record retrieval was not restricted to the publication date. These bibliographic databases were searched on July 31, 2023, and updated twice on February 29, 2024 and October 31, 2024. The reference lists of included articles and relevant reviews were also screened.

### 2.2 Study Selection

Studies from the search strategy were included in the Rayyan app [26] to remove duplicates. Two blinded investigators (S. C. A. and R. C. S.) screened the titles and abstracts. After the initial screening, the full texts were assessed against the inclusion and exclusion criteria by the same blinded researchers. Disagreements were resolved by a third investigator (V. M. G. O. A.). Articles published in English, Spanish, or Portuguese were selected, with this criterion applied during the review of titles and abstracts.

Eligible studies met the following PICOS criteria: (1) Population: patients hospitalized in a PICU using IMV (aged over 28 days up to 18 incomplete years; patients with tracheostomy were not included), (2) Intervention: use of weaning IMV protocol, (3) Comparison intervention: usual weaning care based on the clinician's judgment, (4) Outcome: any primary or secondary outcome (detailed below), and (5) Study design: randomized clinical trials (RCTs), including cluster‐randomized trials.

We chose to exclude neonates because weaning is performed differently in this population [22, 27]. Due to neonatal physiology, the spontaneous breathing test is less predictive, as extubation failure may result from central apnea, atelectasis, or upper airway obstruction rather than the classic markers of respiratory fatigue used in older patients [27]. In contrast, pediatric patients, with more mature respiratory mechanics, may benefit from standardized, protocolized weaning with daily Spontaneous Breathing Trial (SBT), which can more reliably predict readiness for extubation [28, 29].

### 2.3 Outcomes

The study′s primary outcome was the duration of IMV (in days) from tracheal intubation to successful extubation. Secondary outcomes included the LOS in the PICU and hospital (both measured in days), extubation failure (return to IMV through the endotracheal tube within 48 h after withdrawal) [30], and hospital mortality rates.

### 2.4 Data Abstraction

Two authors (S. C. A. and R. C. S.) independently extracted data using structured data collection forms pretested and validated by the review team (File S1). The review team was consulted in cases of discrepancies. The corresponding authors were contacted for further details in case of unclear or unreported data.

For our outcomes of interest, we extracted sample sizes, means, standard deviations (SDs), range scores, interquartile ranges, and confidence intervals (CIs) from all groups. Results reported as medians and interquartile ranges were transformed into means and SDs following the Cochrane recommendations [24] and Wan's method [31].

### 2.5 Risk of Bias

To assess the risk of bias, three reviewers (S. C. A., R. C. S., and I. G. A.) independently evaluated the selected studies using the revised Cochrane Risk of Bias Tool for Randomized Trials (RoB 2) [24]. Any disagreements were resolved through consultation with a fourth author (I. S. S.). The assessment was performed at the primary outcome level. The following sources of bias were evaluated for each trial: the randomization process, deviations from intended interventions, missing outcome data, measurement of the outcome, and selection of the reported result. For cluster‐randomized trials, an additional domain was assessed: bias arising from identifying or recruiting individual participants within clusters. The overall risk‐of‐bias judgment was categorized as low risk of bias, some concerns, or high risk of bias [24].

### 2.6 Statistical Analysis

Statistical analyses were performed using the Cochrane Collaboration Review Manager Web [32]. Mean difference (MD) for continuous data and relative risk (RR) for dichotomous data with 95% CI were computed. We would have used an inverse variance fixed‐effects model for the analyses and a sensitivity analysis with a random‐effects model in cases of unexplained heterogeneity. A heterogeneity analysis was planned using the *χ* ^2^ test and the *I* ^2^ statistic. If significant unexplained heterogeneity had been identified (*χ* ^2^ *p* value of < 0.10 or *I* ^2^ value of > 50%) [33], we would have reported it and explored it through prespecified subgroup analyses. A sensitivity analysis was planned to examine the impact of removing studies with some concerns regarding RoB 2 on the pooled results. However, since most of the analyses were based on data from a single or two studies, we did not perform sensitivity analyses. Protocol deviations are reported in Table S2.

Due to the diverse study designs and variations in weaning protocols—both in type and method of application—we chose to analyze some data separately based on the protocol used. We presented data from studies with different protocols in a forest plot to facilitate visual interpretation. However, we did not pool the data, meaning that the meta‐analysis diamond was disabled when only one study was presented or when heterogeneity was high.

### 2.7 Assessing the Quality of Evidence

The Grading of Recommendations Assessment, Development, and Evaluation (GRADE) approach [34] was applied to assess the quality of evidence for each type of protocol and outcome. The evidence was categorized as very low, low, moderate, or high based on study limitations (risk of bias), imprecision, inconsistency, indirectness, publication bias, and large magnitude of effect [35].

## 3 Results

### 3.1 Systematic Search

**Figure 1:**
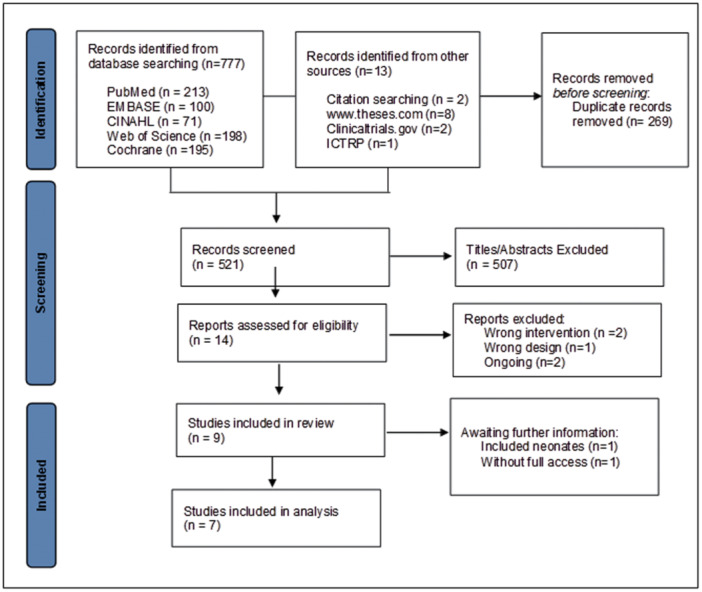
Flow diagram for the selection of studies. ICTRP = World Health Organization International Clinical Trials Registry Platform. [Color figure can be viewed at wileyonlinelibrary.com]

The systematic search identified 790 studies, of which 521 remained after removing duplicates. Title and abstract screening resulted in 14 reports assessed for eligibility, of which 5 were excluded: 3 trials did not meet the review inclusion criteria, and 2 were ongoing (Tables S3 and S4). All identified studies were randomized clinical trials published in English, and consequently, no articles were excluded based on language. Nine studies met the eligibility criteria, but two trials were excluded from the analysis due to insufficient information (Table S5). Therefore, seven trials were included in the final analysis. The PRISMA diagram is presented in Figure 1.

### 3.2 Characteristics of Included Studies

The seven eligible studies [26, 27, 28, 29, 30, 31, 32, 33, 34, 35, 36, 37, 38, 39, 40, 41, 42] published between 2007 and 2021 included 11,742 patients. We have provided a descriptive table (Table 1) summarizing each study's key characteristics.

**Table 1:** Studies characteristics (alphabetical order).

Study	Country	Design	*n*	Inclusion	Protocol group	Control group	Outcomes relevant to this review
Blackwood et al. [36]	UK	Multicenter‐ cluster (17 hospital sites sequentially, all sites did intervention and control)	8828	Infants and children (aged < 16 years) requiring IMV	Education and training for the multiprofessional PICU team to deliver an assessment of sedation levels using COMFORT scale scores, daily screening for readiness to undertake an SBT, initiation of an SBT when screening criteria were satisfied, and a daily multiprofessional round.	Usual care: slow reduction in ventilator support to low levels of support before extubation, without sedation protocol or ventilator liberation protocols.	Duration of IMV; Successful extubation until 48 h; PICU and hospital LOS; In‐hospital mortality.
Curley et al. [37]	USA	Multicenter‐ cluster (17 sites for intervention and 14 sites for control)	2449^a^	Patients 2 weeks to 17 years of age requiring IMV for acute airways and/or parenchymal lung disease^a^	Training for the interprofessional PICU team to deliver sedation using a protocol that included targeted sedation, arousal assessments, sedation adjustment every 8 h, sedation weaning, and extubation readiness testing.	Usual care for managed sedation and weaning, without a protocol or recommendations for extubation readiness testing.	Duration of IMV; PICU and hospital LOS; In‐hospital mortality; Reintubation within 24 h.
Foronda et al. [38]	Brazil	Multicenter‐ two hospitals (children were randomized after intubation)	260	Children 28 days to 15 years of age requiring IMV	Weaning protocol combining daily evaluation to check readiness for weaning with a 2‐h spontaneous breathing test.	Standard care procedures (did not include daily screening or a spontaneous breathing test). The ventilator mode and settings were at the discretion of the attending physician.	Duration of IMV; Extubation failure; Mortality in the PICU.
Jouvet et al. [39]	Canada	Single center. Children were randomized after assessment of weaning criteria and spontaneous breathing test.	30	Children 2–18 years, body weight ≥ 15 kg admitted to the PICU, requiring IMV.	Automated weaning protocol using SmartCare/PSTM of Evita XL respirator.	Wean according to physicians practice, without protocol (assessment of weaning criteria and spontaneous breathing test with pressure support was performed).	Duration of IMV; Reintubation within 48 h; PICU and hospital LOS; Mortality in the PICU.
Keivanfar et al. [40]	Iran	Single center. Children were randomized after intubation	68	Children in PICU requiring IMV	Protocolized respiratory care: daily assessment of arterial blood gas test to reduce ventilatory parameters, evaluation of readiness criteria, and spontaneous breathing test.	The attendant decides on reducing parameters and extubation according to the results of arterial blood gas tests and based on the patient's clinical conditions.	Duration of IMV; Reintubation; PICU and hospital LOS; Mortality in the PICU.
Kishore and Jhamb [41]	India	Single center. Children were randomized after passing on eligibility criteria for weaning.	76	Children 1 month to 12 years of age in PICU, requiring IMV.	Weaning protocol composed of daily evaluation of eligibility criteria for weaning and spontaneous breathing test (pressure support and T‐piece).	Conventional physician‐directed weaning (included eligibility criteria, gradual reduction of ventilatory parameters, and spontaneous breathing test with T‐piece).	Duration of IMV; PICU and hospital LOS; Reintubation within 48 h.
Maloney [42]	USA	Single center. Children were randomized after two consecutive decreases in Volume tilde, PEEP, mechanical RR or PS had occurred.	31	Children in the PICU requiring IMV for acute respiratory failure from intrinsic lung disease.	Specific instructions to decrease ventilatory support until the patient was extubated or failed a weaning trial, using a computerized ventilator weaning protocol (Java platform and Blaze Advisor rules described fully in the thesis).	Weaning without a protocol. The care team used their clinical judgment to determine the time to initiate ventilator weaning, reduce parameters, and extubate.	Duration of IMV; PICU and hospital LOS; Reintubation within 36 h.

Abbreviations: IMV, invasive mechanical ventilation; LOS, length of stay; PEEP, positive end‐expiratory pressure; PICU, Pediatric Intensive Care Unit; PS, pressure support; RR, respiratory rate; UK, United Kingdom; USA, United States of America.

a Total study sample. In the meta‐analysis, data from neonates and tracheostomized patients were removed, according to the data spreadsheet provided by the author (*n* = 2349).

The median age ranged from 1.4 to 114.6 months (only one study reported age data as a mean, ranging from 116 to 119 months). In the study that included patients younger than 29 days, we contacted the author to exclude the neonatal data [37].

Two studies included only respiratory distress as an indication of intubation [37, 38, 39, 40, 41, 42]. The other 5 studies included more indications for intubation due to neurological disease, cardiovascular disease, postoperative status, altered mental state, gastroenterological disease, and others (see Table S6).

### 3.3 Interventions and Comparisons

In the intervention group, two studies focused on training the interprofessional PICU team in a sedation weaning protocol [36, 37]. One of them assessed sedation levels using the COMFORT scale score and implemented a ventilation weaning protocol with daily readiness screening and a spontaneous breathing test [36]. Another study used a goal‐directed algorithm to guide sedation therapy, setting a target score in the State Behavioral Scale and adjusting sedatives based on the illness phase. It also incorporated ventilation weaning with an extubation readiness test [37]. Two studies applied the ventilation weaning protocol, one included daily readiness screenings and spontaneous breathing test [38], and another included reduction of ventilatory parameters through daily blood gas analysis, assessment of weaning criteria, and spontaneous breathing test [40]. Two studies implemented a computerized automated ventilator weaning protocol [39, 42]. One study implemented a weaning protocol that included a daily assessment of weaning criteria and two spontaneous breathing tests: pressure support and the T‐piece [41]. Notably, in this study, the key difference from the control group was the use of the spontaneous breathing test with pressure support [41]. The characteristics of each protocol are attached to Table S7.

For the control group, five studies applied usual care for sedation management and ventilator weaning, without a protocol or spontaneous breathing tests, relying instead on the clinical judgment of the team or treating clinician for ventilator weaning [36, 37, 38, 40, 42]. One study followed ventilator weaning based on physicians' practices without a protocol, but conducted a spontaneous breathing test before randomization [39]. Another study applied daily readiness screening, like the intervention, and a spontaneous breathing test with a T‐piece, as it was already standard practice in the PICU [41] (see Table 1).

### 3.4 Risk of Bias

**Figure 2:**
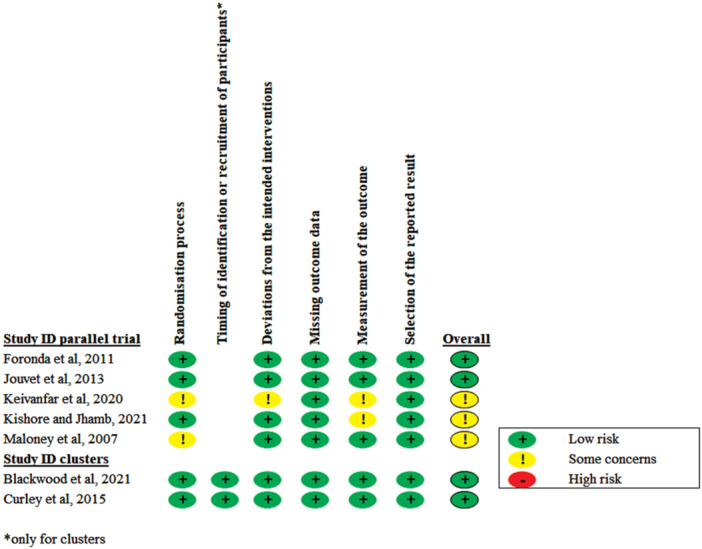
Risk of bias. [Color figure can be viewed at wileyonlinelibrary.com]

Four studies [36, 37, 38, 39] were deemed to have a low RoB 2 in all domains. Three studies raised some concerns in at least one domain: two in allocation concealment [40, 42], one in deviation from intended intervention [40], and two in outcome measurement [40, 41] (see Figure 2 and Table S8). Details regarding funding sources and author conflicts of interest for the included studies are provided in Table S9.

### 3.5 Effects of Weaning Protocol

All included studies reported the IMV time outcome; three presented it in hours [36, 39, 42], which was transformed into days to compare and provide more reliable data. Six studies reported the LOS in the PICU and hospital [36, 37, 39, 40, 41, 42]; one study presented this outcome in hours [42], which were transformed into days. All included studies were reported in the analysis of extubation failure [36, 37, 38, 39, 40, 41]; however, two of them defined extubation failure differently from the criteria adopted in this review [37, 42]. Therefore, we conducted a sensitivity analysis, which showed no significant change in the results when these studies were excluded (see Table S10). The hospital mortality outcome was reported in two studies [36, 37].

Five studies excluded patients who died from their analyses [36, 38, 40, 41, 42]. In two studies, it was not reported whether such patients were excluded; however, they used the ventilator‐free days [37, 39].

Due to significant differences in methodology and the protocols used in the studies, a meta‐analysis could not be conducted for all outcomes and analyses. Therefore, the analysis was performed separately according to the protocol used, as studies employing similar protocols shared comparable methodologies. Given the substantial heterogeneity observed in some of the analyzed outcomes, when possible, we conducted a subgroup analysis based on the cause of intubation, distinguishing between studies including children intubated for diverse reasons and those limited to respiratory disease.

The certainty of evidence was reported alongside each estimate for each protocol and is presented in the summary of findings (Table 2).

**Table 2:** Summary of findings—types of protocol versus usual care in Pediatric Intensive Care Unit (PICU).

	**Anticipated absolute effects** * **(95% CI)**					
**Outcomes**	**Risk with usual care**	**Risk with weaning sedation and ventilation protocol**	**Relative effect (95% CI)**	**No. of participants (studies)**	**Certainty of the evidence (GRADE)**	**Comments**
*1. Weaning sedation plus ventilation protocol compared to usual care*						
*Duration of IMV*		MD *0.02 days higher* (0.12 lower to 0.17 higher)	—	11,188 (2 RCT)	⊕⊕⊕◯ Moderate^b^	There may be no clinically important difference
*PICU LOS*—intubated for a variety of reasons		MD *0.33 days higher* (0.13 higher to 0.53 higher)	—	8843 (1 RCT)	⊕⊕⊕◯ Moderate^a^^,^ c	
*PICU LOS*—intubated for respiratory disease		MD *0.24 days lower* (0.88 lower to 0.4 higher)	—	2199 (1 RCT)	⊕⊕⊝⊝ Low^a^^,^ b	
*Hospital LOS*—intubated for a variety of reasons		MD *0.47 days higher* (0.01 lower to 0.95 higher)	—	7591 (1 RCT)	⊕⊕⊝⊝ Low^a^^,^ b	
*Hospital LOS—*intubated for respiratory disease		MD *2.33 days lower* (3.42 lower to 1.24 lower)	—	2199 (1 RCT)	⊕⊕⊕◯ Moderate^a^^,^ c	The weaning sedation plus ventilation protocol probably decreases hospital LOS in children intubated for respiratory disease
*Extubation Failure*	114 per 1000	*108 per 1000* (97–120)	*RR 0.95* (0.85–1.05)	11,192 (2 RCT)	⊕⊕⊕◯ Moderate^b^^,^ c	There may be no clinically important difference
*Hospital mortality* intubated for a variety of reasons	53 per 1000	*63 per 1000* (52–75)	*RR 1.19* (0.99–1.42)	8063 (1 RCT)	⊕⊕⊝⊝ Low^a^^,^ b	The hospital mortality may be higher in the weaning sedation plus ventilation protocol in children intubated for a variety of reasons
*Hospital mortality* intubated by respiratory disease	70 per 1000	*57 per 1000* (42–78)	*RR 0.81* (0.60–1.11)	2349 (1 RCT)	⊕⊕⊝⊝ Low^a^^,^ b	The weaning sedation plus ventilation protocol may decrease hospital mortality in children intubated by respiratory disease
*2. Weaning protocol (daily screening criteria plus spontaneous breathing test) compared to usual care*						
*Duration of IMV*		MD *1.2 days lower* (1.27 lower to 1.13 lower)	—	260 (1 RCT)	⊕⊕⊕◯ Moderate^a^^,^ c	Weaning protocol probably decreases IMV time
*Duration of IMV* **		MD *1.67 days higher* (0.01 higher to 3.33 higher)	—	55 (1 RCT)	⊕⊝⊝⊝ Very low^a^^,^ d ^,^ e	The certainty of the evidence was too low for conclusions
*PICU LOS* **		MD *1.77 days higher* (1.58 lower to 5.12 higher)	—	68 (1 RCT)	⊕⊝⊝⊝ Very low^a^^,^ d ^,^ e	
*Hospital LOS* **		MD *2.67 days higher* (2.95 lower to 8.29 higher)	—	68 (1 RCT)	⊕⊝⊝⊝ Very low^a^^,^ d ^,^ e	
*Extubation Failure*	138 per 1000	*102 per 1000* (56–184)	*RR 0.74* (0.41–1.34)	328 (2 RCT)	⊕⊕⊕◯ Moderate^b^^,^ f	The weaning protocol probably decreases extubation failure
*3. Automated computer‐driven weaning protocol compared to usual care*						
*Duration of IMV*		MD *1.59 days lower* (3.09 lower to 0.09 lower)	—	61 (2 RCTs)	⊕⊕⊝⊝ Low^f^^,^ g	Automated computer‐driven weaning protocol may decrease IVM time
*PICU LOS*		MD *3.83 days lower* (9.66 lower to 2.00 higher)	—	61 (2 RCTs)	⊕⊝⊝⊝ Very low^f^^,^ h ^,^ i	The certainty of the evidence was too low for conclusions
*Hospital LOS*		MD *4.51 days lower* (10.87 lower to 1.86 higher)	—	61 (2 RCTs)	⊕⊝⊝⊝ Very low^f^^,^ h	
*Extubation Failure*	97 per 1000	*132 per 1000* (32–551)	*RR 1.36* (0.33–5.69)	61 (2 RCT)	⊕⊝⊝⊝ Very low^h^	
*4. Daily readiness criteria plus spontaneous breathing test by pressure support and T‐piece test versus daily readiness criteria plus T‐piece test*						
*Duration of IMV*		MD *1.06 days lower* (3.39 lower to 1.27 lower)	—	76 (1 RCTs)	⊕⊝⊝⊝ Very low^a^^,^ d ^,^ e	The certainty of the evidence was too low for conclusions
*PICU LOS*		MD *2.90 days higher* (4.99 lower to 10.79 higher)	—	76 (1 RCTs)	⊕⊝⊝⊝ Very low^a^^,^ d ^,^ e	
*Hospital LOS*		MD *0.94 days lower* (11.84 lower to 9.96 higher)	—	76 (1 RCTs)	⊕⊝⊝⊝ Very low^a^^,^ d ^,^ e	
*Extubation Failure*	132 per 1000	*105 per 1000* (30–362)	*RR 0.80* (0.23–2.75)	76 (1 RCTs)	⊕⊝⊝ Very low^a^^,^ d ^,^ e	

*Note:* GRADE Working Group grades of evidence: *High certainty:* we are very confident that the true effect lies close to that of the estimate of the effect. *Moderate certainty:* we are moderately confident in the effect estimate: the true effect is likely to be close to the estimate of the effect, but there is a possibility that it is substantially different. *Low certainty*: our confidence in the effect estimate is limited: the true effect may be substantially different from the estimate of the effect. *Very low certainty:* we have very little confidence in the effect estimate: the true effect is likely to be substantially different from the estimate of effect.

Abbreviations: CI, confidence interval; MD, mean difference; RR, risk ratio.

* *The risk in the intervention group* (and its 95% confidence interval) is based on the assumed risk in the comparison group and the *relative effect* of the intervention (and its 95% CI).

** In addition to the weaning protocol, this study also included the gradual reduction of ventilatory support to minimum settings.

a The evidence was downgraded for inconsistency due to the reliance on a single study, which prevented a heterogeneity analysis.

b Optimal information size criterion reached; we downgraded the evidence because CIs included both an effect and no effect.

c Optimal information size criterion reached.

d The evidence was downgraded because there were some concerns about assessing the risk of bias.

e The evidence was downgraded twice for serious imprecision due to a small sample size, and the CIs included both an effect and no effect.

f There was a study that raised some concerns about assessing the risk of bias. Potential limitations are unlikely to diminish confidence in the effect estimate.

g The evidence was downgraded twice for imprecision due to the small sample size and the fact that one of the studies was a feasibility study.

h The evidence was downgraded three times for imprecision due to the small sample size, because the CIs included both an effect and no effect, and the fact that one of the studies was a feasibility study.

i There was substantial variability in effect estimates (*I* ^2^ = 58% and *p* value for heterogeneity = 0.12).

#### 3.5.1 Primary Outcome—Duration of IMV

**Figure 3:**
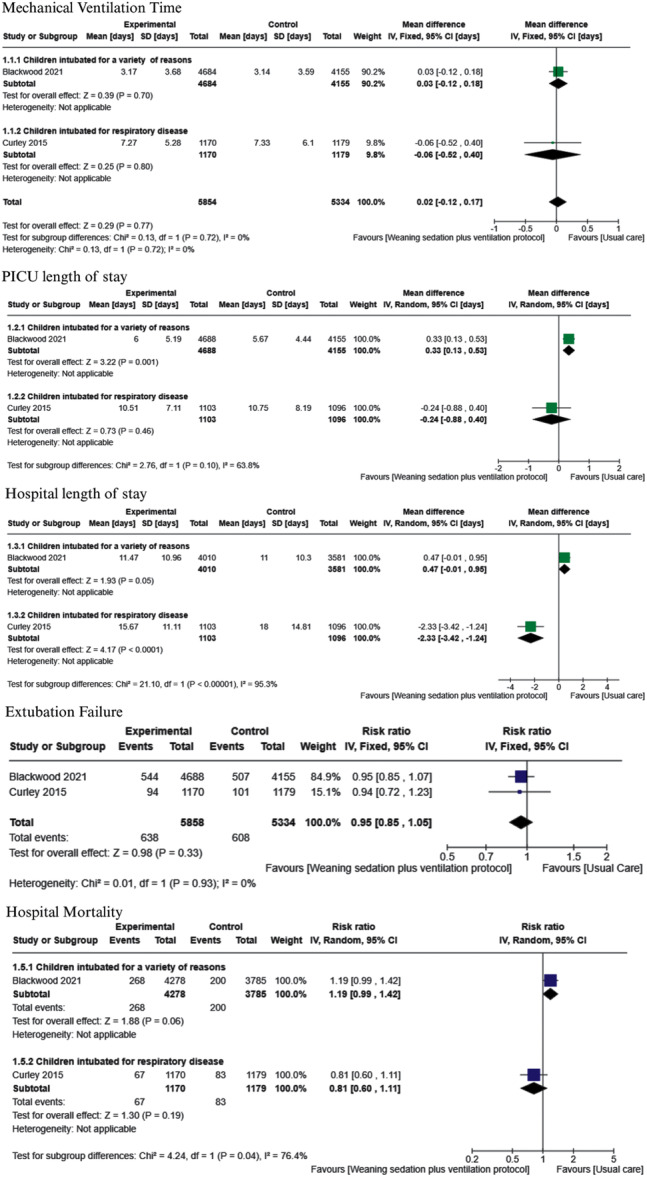
Weaning sedation plus ventilation protocol versus usual care. [Color figure can be viewed at wileyonlinelibrary.com]

Two trials incorporated a combined sedation and ventilation weaning protocol [36, 37], with the corresponding analysis presented in Figure 3. In this type of protocol, the duration of IMV presented no difference between weaning sedation plus ventilation protocol versus usual care (MD 0.02, 95% CI −0.12 to 0.17; 2 trials, *N* = 11,188; moderate‐certainty evidence).

**Figure 4:**
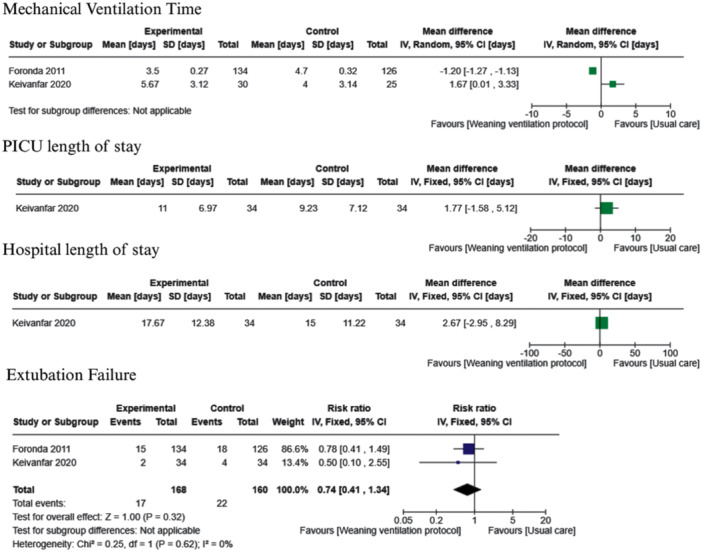
Weaning protocol (daily screening criteria + spontaneous breathing test) versus usual care. [Color figure can be viewed at wileyonlinelibrary.com]

Two studies implemented a weaning protocol consisting of daily screening criteria followed by a spontaneous breathing test [38, 40], with the corresponding analysis presented in Figure 4. Foronda et al. [38] conducted a weaning protocol that included daily assessments of weaning criteria and spontaneous breathing tests with pressure support. In contrast, Keivanfar et al. [40] implemented a gradual reduction of ventilatory parameters to minimum levels before initiating the evaluation of weaning criteria. When we performed a meta‐analysis of these two trials, substantial heterogeneity was observed; therefore, we decided to report the analysis separately. In the weaning protocol by Foronda et al. [38], IMV duration decreases with the weaning protocol compared to usual care (MD −1.2, 95% CI −1.27 to −1.13; 1 trial, *N* = 260; moderate‐certainty evidence). However, in the weaning protocol by Keivanfar et al. [40], there was no clear difference between using a weaning protocol compared to usual care (MD 1.67, 95% CI 0.01–3.33; 1 trial, *N* = 55; very low‐certainty evidence).

**Figure 5:**
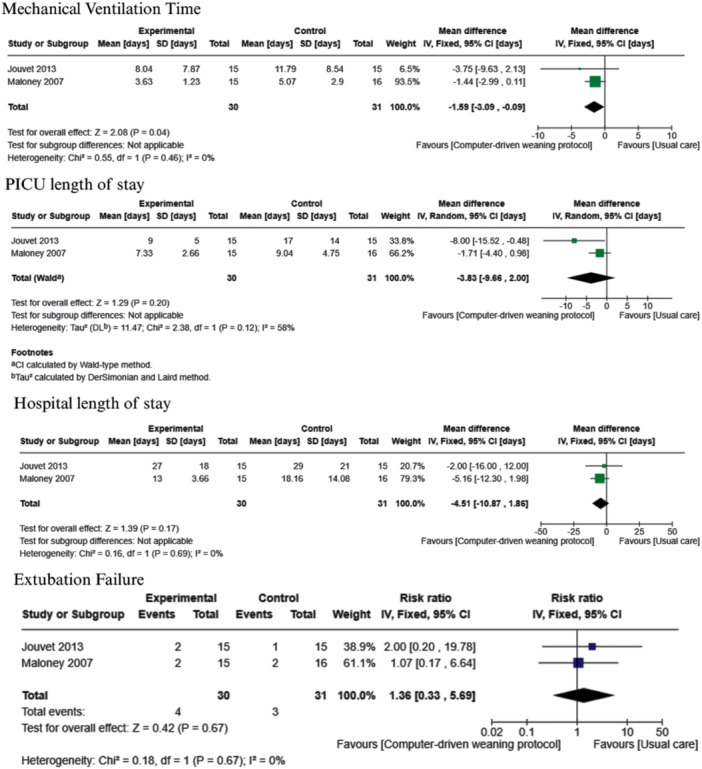
Computer‐driven weaning protocol versus usual care. [Color figure can be viewed at wileyonlinelibrary.com]

Two studies implemented the automated computer‐driven weaning protocol [39, 42], with the corresponding analysis presented in Figure 5. This weaning protocol may decrease IMV duration compared to usual care (MD −1.59, 95% CI −3.09 to −0.09; 2 trials, *N* = 61; low‐certainty evidence).

**Figure 6:**
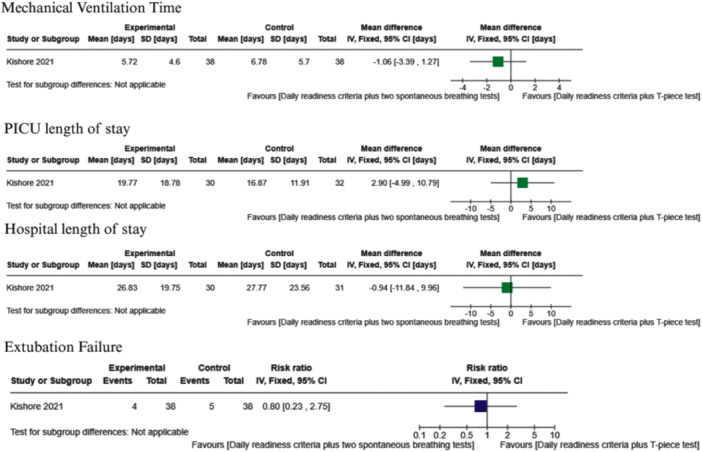
Daily readiness criteria plus two spontaneous breathing tests by pressure support and T‐piece test versus Daily Readiness criteria plus Spontaneous breathing test by T‐piece. [Color figure can be viewed at wileyonlinelibrary.com]

One study implemented a weaning protocol consisting of daily readiness criteria plus two spontaneous breathing tests (pressure support and T‐piece tests) compared to daily readiness criteria plus a spontaneous breathing test by T‐piece [41], with the corresponding analysis presented in Figure 6. It is uncertain whether the implemented intervention improves IMV duration (MD −1.06, 95% CI −3.39 to 1.27; 1 trial, *N* = 76; very low‐certainty evidence).

#### 3.5.2 Secondary Outcomes—PICU and Hospital LOS

The weaning sedation plus ventilation protocol [36, 37] exhibited substantial heterogeneity (*I* ^2^ > 50%); therefore, we conducted a subgroup analysis stratified by intubation cause. Data were represented in a forest plot for visual interpretation (Figure 3). Among children intubated for a variety of reasons [36], there was no clinically meaningful difference between the weaning sedation plus ventilation protocol and usual care in PICU LOS (MD 0.33, 95% CI 0.13–0.53; *N* = 8843; moderate‐certainty evidence) or hospital LOS (MD 0.47, 95% CI −0.01 to 0.95; *N* = 7591; low‐certainty evidence). For children intubated for respiratory disease [37], PICU LOS was similar between groups (MD −0.24, 95% CI −0.88 to 0.40; *N* = 2199; low‐certainty evidence), while hospital LOS was 2.33 days shorter in the weaning protocol group compared with usual care (MD −2.33, 95% CI −2.42 to −1.24; *N* = 2199; moderate‐certainty evidence).

For the weaning protocol involving daily screening criteria followed by a spontaneous breathing test, only one study assessed PICU and hospital LOS [40]. The data were represented in a forest plot for visual interpretation (Figure 4). No clear differences were observed between groups for PICU LOS (MD 1.77, 95% CI −1.58 to 5.12; *N* = 68; very low‐certainty evidence) or hospital LOS (MD 2.67, 95% CI −2.95 to 8.29; *N* = 68; very low‐certainty evidence).

The effectiveness of the automated computer‐driven weaning protocol [39, 42] on PICU and hospital LOS remains highly uncertain due to the very low certainty of the available evidence. Results from two small trials (*N* = 61) suggest a possible reduction in PICU LOS (MD −3.83, 95% CI −9.66 to 2.00) and hospital LOS (MD −4.51, 95% CI −10.87 to 1.86), although the CIs are wide and overlap the null effect. These findings are summarized in Figure 5.

The effect of daily readiness criteria combined with two spontaneous breathing tests (pressure support and T‐piece) compared with daily readiness criteria plus a single T‐piece spontaneous breathing test [41] on clinical outcomes remains uncertain. Evidence from one small trial suggests no clear difference in PICU LOS (MD 2.90, 95% CI −4.99 to 10.79; *N* = 62) or hospital LOS (MD −0.94, 95% CI −11.84 to 9.96; *N* = 61). However, the certainty of the evidence is very low. These findings are summarized in Figure 6.

#### 3.5.3 Secondary Outcomes—Extubation Failure

The weaning sedation plus ventilation protocol [36, 37] in the extubation failure outcome is presented in Figure 3. There was no clinically meaningful difference between groups (RR 0.95, 95% CI 0.85–1.05; 2 trials, *N* = 11,192; moderate‐certainty evidence).

For the weaning protocol involving daily screening criteria followed by a spontaneous breathing test, data from two trials (*N* = 328) [38, 40] suggest a potential 26% reduction in extubation failure in the weaning protocol group (RR 0.74, 95% CI 0.41–1.34; moderate‐certainty evidence); however, the CI overlaps the null effect.

Evidence on the effectiveness of automated computer‐driven weaning protocols for extubation failure is very uncertain. No clinically meaningful difference was found between groups (RR 1.36, 95% CI 0.33–5.69; 2 trials, *N* = 61; very low‐certainty evidence). The wide CI leaves the findings inconclusive (Figure 5).

The effectiveness of combining daily readiness criteria with two spontaneous breathing tests (pressure support and T‐piece) compared with daily readiness criteria plus a single T‐piece test [41] in reducing extubation failure remains uncertain. Evidence from one trial (*N* = 76) suggested a potential reduction in risk (RR 0.80, 95% CI 0.23–2.75); however, the CI includes the null effect, and the certainty of the evidence is very low. These findings are summarized in Figure 6.

#### 3.5.4 Secondary Outcomes—Hospital Mortality

The weaning sedation plus ventilation protocol [36, 37] showed substantial heterogeneity (*I* ^2^ > 50%); therefore, we performed a subgroup analysis stratified by intubation cause, with results displayed in a forest plot for visual interpretation (Figure 3). Among children intubated for a variety of reasons [36], the protocol suggested a tendency toward a 19% increase in hospital mortality compared with usual care (RR 1.19, 95% CI 0.99–1.42; 1 trial, *N* = 8063; low‐certainty evidence); however, the CI overlapped the null effect, indicating uncertainty in the estimate. In contrast, in children intubated for respiratory disease [37], the protocol suggested a tendency toward a 19% reduction in hospital mortality (RR 0.81, 95% CI 0.60–1.11; 1 trial, *N* = 2349; low‐certainty evidence), though again the CI overlapped the null effect, indicating uncertainty in the estimate. This outcome was not reported in the remaining studies.

## 4 Discussion

### 4.1 Summary of Main Results

This systematic review evaluated the effects of implementing a weaning protocol compared to usual care in the PICU. Nine studies satisfied the inclusion criteria, but only seven could be included in the final analysis. Due to methodological differences and variations in the protocols used across studies, a meta‐analysis including all studies and outcomes was not feasible. Therefore, we opted to analyze the data separately based on the type of protocol applied.

#### 4.1.1 Primary Outcome

Significant reductions in mechanical ventilation duration were observed only with the weaning protocol based on daily assessment of weaning criteria and a spontaneous breathing test using pressure support (1.2 days; moderate‐certainty evidence) [38] and with computer‐automated weaning (1.5 days; low‐certainty evidence) [39, 42]. However, another study [40] that implemented a weaning protocol based on a daily assessment of weaning criteria and a spontaneous breathing test with pressure support did not show a difference between the weaning protocol and usual care, with very low certainty of evidence. The difference in results between the weaning protocols may be explained by the fact that this study reduced ventilatory parameters to minimal levels before assessing weaning readiness, which prolonged IMV duration.

The weaning sedation plus ventilation protocol presented no differences in IMV duration between the protocol and the usual care group, with moderate certainty of evidence [36, 37]. Data from the Blackwood et al. study [36] used in the analysis were based on the raw IMV time reported in the article, as the authors were contacted, but the original data set could not be obtained. However, the authors reported a significant reduction in IMV time in the sedation and ventilation weaning group when the outcomes were adjusted for a cluster (PICU) and calendar time (period categorical effect), reporting a median difference of −6.1 h (−8.2 to −5.3) with *p* = 0.02. The 6‐hour difference was statistically significant but may not be clinically relevant, as prior studies have generally considered differences exceeding 1 day in IMV duration to be clinically relevant [37, 38, 43, 44].

#### 4.1.2 Secondary Outcomes

Analysis of the sedation plus ventilation weaning protocol demonstrated substantial heterogeneity in secondary outcomes. To address this issue, we performed a subgroup analysis by cause of intubation. Including patients with different causes of intubation increases heterogeneity, as the underlying condition, its severity, and the time required for resolution can influence the duration of mechanical ventilation, length of hospital stay, and mortality. The weaning sedation plus ventilation protocol in children intubated for respiratory disease reported a significant reduction in the LOS for 2 days in the hospital in the protocol group [37]. In contrast, the other protocols did not show significant differences.

About the extubation failure outcome, the present review defined it as reintubation within 48 h. All studies analyzed the extubation failure outcome, but one considered the use of noninvasive ventilation as a failure [40], which also presented results from reintubation in 48 h and could be included in the analysis. Two studies considered extubation failure by reintubation in 24 [37] and 36 h [42], which were included in the analysis. To understand the influence of this difference on the results, a sensitivity analysis was performed, in which no differences were observed when these studies were removed from the analyses. The weaning protocol based on daily assessment of weaning criteria combined with a spontaneous breathing test using pressure support showed a trend toward a clinically relevant 26% reduction in extubation failure, with moderate‐certainty evidence [38, 40]. However, the wide CIs overlapped with the null effect. These two studies [38, 40] demonstrated substantial heterogeneity for IMV duration but were homogeneous for extubation failure. Notably, reducing parameters to minimal levels before meeting daily criteria did not influence extubation failure, as it did IMV duration. No other protocols demonstrated differences in extubation failure.

Another key outcome of protocol use in the PICU is mortality. Hospital mortality was reported in only two studies comparing the weaning sedation and ventilation protocol with usual care [36, 37]. No significant differences were observed between protocol and usual care, as the CIs overlapped the null effect.

### 4.2 Differences Among Studies and Heterogeneity Analysis

As shown in Table S7, there was considerable variation in the implementation of weaning protocols across pediatric ICUs. In some studies, sedation weaning was part of the ventilator weaning [36, 37], whereas others focused on evaluating weaning criteria and conducting a SBT [38, 40]. One study [40] implemented a gradual reduction of ventilatory parameters to minimal settings before assessing readiness to wean—a strategy that may unnecessarily prolong mechanical ventilation, as not all pediatric patients require extubation at minimal parameters. Other approaches included computer‐automated weaning [39, 42], while another study compared two SBT techniques (T‐piece vs. pressure support) against the T‐piece alone.

Substantial differences were also observed in patient populations: some studies included only respiratory patients, whereas others encompassed a broader range of conditions such as cardiovascular and neurological disorders, trauma, cardiac arrest, sepsis, and postoperative cases. Age criteria likewise varied, with inclusion ranging from 28 days to 18 years. One study set the lower age limit at 2 years [38], while most studies used an age range of 28–30 days; upper age limits ranged from 15 to 18 years. Notably, no study stratified outcomes by age group, and only one author provided stratified data [37]. Therefore, subgroup analysis by age could not be performed.

Methodological variability was also evident. Protocols involving both sedation and ventilation weaning were randomized at the PICU level, while those evaluating weaning criteria and SBTs were randomized at the patient level. Automated weaning protocols were tested in one pilot randomized study with a small sample size and in one early randomized study with similarly limited numbers. Finally, a single trial applied the same protocol to both groups, differing only in the SBT technique evaluated.

Given these differences, when all studies were pooled in the meta‐analysis, substantial heterogeneity was observed. Therefore, the analysis was conducted separately according to the type of protocol, as studies employing similar protocols shared comparable methodologies. The limited number of studies precluded additional subgroup analyses.

### 4.3 Weaning Protocol: Current Practices and Evidence Gaps

Our analysis of the protocols applied across the included studies revealed substantial heterogeneity in weaning techniques, mirroring the variability observed in clinical practice within PICU. This variation has also been confirmed in previous survey studies [22, 23, 45]. Notably, all studies were conducted before the publication of the 2023 Ventilator Liberation Guidelines in Children [46]. Future research should align with these guidelines to promote greater standardization of care.

The recent Ventilator Liberation Guidelines in Children recommend assessing eligibility for extubation readiness testing (ERT) through scheduled evaluations of physiological, ventilatory, or disease‐specific milestones [46]. They also suggest using an ERT bundle, involving daily screening of sedation depth, airway control, risk of post‐extubation obstruction, respiratory muscle strength, secretion burden, hemodynamic stability, and post‐extubation support planning, to guide liberation from IMV in pediatric patients [46].

All studies in this review conducted daily weaning readiness assessments, but the specific criteria varied across studies. This variability highlights the need to standardize assessment criteria in both research and clinical practice. The Ventilator Liberation Guidelines in Children [46] provides suggested items for inclusion in extubation readiness assessments. Several studies have proposed using bundles for daily readiness assessment [28, 47, 48].

The most recent guidelines recommend incorporating an SBT into the ERT bundle to evaluate a child's ability to sustain adequate ventilation and gas exchange [46]. Among the studies included in this review, only one did not perform an SBT [42]. Most used pressure support mode [37, 38, 39, 40, 41], while a single study applied continuous positive airway pressure (CPAP) [36]. The guideline advises using pressure support with CPAP, or CPAP alone in patients at high risk of extubation failure; however, this recommendation is based on very low‐certainty evidence [46], derived from one randomized trial showing no significant difference between pressure support and T piece test [49], and observational studies suggesting that pressure support may underestimate post‐extubation respiratory effort [50, 51, 52]. In this review, only one study applied pressure support with CPAP [36], and none stratified protocols according to risk of extubation failure, applying the same approach to all children.

As shown, most recommendations remain ungraded or are supported by low to very low certainty, reflecting the limited number of high‐quality randomized studies demonstrating significant effects [46], a limitation also evident in this review. This review focused on informing clinical practice based on randomized trials. It highlights the scarcity of such trials and underscores that current practice often relies on lower‐quality evidence, emphasizing the need for high‐quality studies.

Notably, studies on computerized weaning were published over a decade ago. Given recent advances in artificial intelligence and machine learning, there is now potential to develop more sophisticated, adaptive weaning algorithms that could improve patient outcomes, optimize ventilator use, reduce IMV duration, and PICU LOS. This highlights the need for renewed investigation into the role of modern computerized systems in ventilator weaning.

### 4.4 Applicability of Evidence

Both the daily assessment of weaning criteria with a spontaneous breathing test using pressure support and the computer‐automated weaning protocol reduced mechanical ventilation duration by more than 1 day. However, the protocols did not demonstrate significant differences in PICU and hospital LOS, extubation failure, or mortality compared with usual care. The type of protocol applied may influence the extent of reduction in IMV duration. Similarly, nonrandomized studies have reported reductions in IMV duration without an associated increase in reintubation rates when protocols were applied [18, 28]. Prolonged IMV is linked to increased complications, adverse events [4, 53], and healthcare costs [54]. Therefore, reducing IMV time by 1 day might improve patient outcomes by decreasing the risk of complications and lowering healthcare costs [55].

### 4.5 Certainty of the Evidence

The certainty of evidence for the sedation plus ventilation protocol was downgraded for imprecision, as CIs included both benefit and no effect for IMV duration and extubation failure. Evidence for hospital LOS for the same protocol was downgraded for inconsistency, as it relied on a single study, preventing heterogeneity analysis. The daily assessment protocol with a spontaneous breathing test [38] was also downgraded for inconsistency in the IMV duration outcome, as it was based on a single study. The weaning protocol involving prior parameter reduction was downgraded for inconsistency, risk of bias, and serious imprecision due to the small sample size and wide CIs. Finally, evidence for the computer‐automated protocol was downgraded twice because of the small sample size and inclusion of a pilot feasibility study [39]. The small sample size of these studies is justified because implementing automated weaning protocols is challenging, requiring specialized ventilators and trained staff [39, 42]. Additionally, Jouvet et al. [39] restricted participant age, as the automated system used in the study is not licensed for children under 2 years old.

### 4.6 Heterogeneity and Challenges in Pediatric Ventilator Weaning

The lack of concrete results on the benefits of using a weaning protocol in pediatrics can be partially explained by the variety of protocols applied and the heterogeneity of the pediatric population. It is also important to consider that the comparison was made against usual care, which varied across studies. In most cases, the usual care involves the clinician or care team making the extubation decision, and in such cases, the clinician could base their decision on established criteria, potentially resembling the protocol [38, 40]. The usual care likely drifted closer to the intervention by protocol. This does not necessarily mean that the weaning protocol was not beneficial.

Ventilator weaning is complex, with heterogeneity arising from differences in population characteristics such as age, diagnoses, and protocols. The pediatric population is diverse, with varying lung physiology between infants, children, and fully developed adolescents and adults [56]. Additionally, the PICU treats a wide range of conditions (respiratory, cardiovascular, neurological, and more) that affect intubation causes and weaning processes [36, 38, 39]. Weaning methods also vary significantly, tailored to the team's expertise and the patient's age, diagnosis, and complications [6, 8]. There were many methodological differences among the studies included in this review, such as the use of automated computer‐driven or professional‐led protocols, types of reduction parameters, types of sedation protocol, the type and number of criteria used to assess readiness to wean, and varying approaches to conducting spontaneous breathing tests, such as duration (30 min or 2 h) and modes (pressure support ventilation or T‐piece).

It was possible to elucidate that the application of the weaning protocol permeates a variety of scenarios within a PICU, spanning different age groups, clinical conditions, diagnoses, and complexities. These variations highlight the need for structured protocols tailored to the specific needs of each population and condition.

### 4.7 Limitations and Strengths

This review has several limitations, primarily related to the heterogeneity of the included trials, such as wide variation in patient age (with outcomes not stratified by age), diverse diagnostic categories, and differing weaning protocols. In addition, only three of the included RCTs were multicenter, and some raised concerns regarding the risk of bias. Furthermore, the trial by Curley et al. [37] relied heavily on benzodiazepine use, which has been associated with a higher incidence of delirium—a condition linked to increased morbidity and mortality [57].

Strengths of this study include its rigorous, systematic approach grounded in Cochrane methodology and the use of a Certainty of Evidence assessment. The review was conducted according to a preregistered protocol, with a comprehensive literature search and duplication of all review processes. Risk of bias was independently assessed by three authors, and data extraction was performed using a pretested, validated form.

## 5 Conclusions

Limited evidence indicates that weaning protocols may shorten IMV duration and that sedation and weaning protocols may reduce hospital stay in children with respiratory disease admitted to the PICU. However, their safety and effectiveness in reducing extubation failure or mortality remain unproven. Due to heterogeneity in the applied protocols and studies' methodologies, the evidence was limited. This underscores the need for high‐quality studies, as current practice is based on low‐certainty evidence.

Greater standardization of weaning protocols in the PICU is needed. Future studies should account for age groups (infants, children, and adolescents) and specific diagnoses, given the heterogeneity of the pediatric population. Protocol development should draw on the best available strategies, leverage current technology, and be tailored to patient needs with clearly defined outcomes.

## Author Contributions

All authors involved in conceptualization, accessibility to data, interpretation of data, and article revision, editing, and approval. Suzana C. Almeida conceived, designed, and drafted the protocol. Suzana C. Almeida and Rayany C. de Souza were involved in search strategy, screened and reviewed, data curation. Suzana C. Almeida, Rayany C. de Souza, and Ingrid G. Azevedo were involved in the risk of bias analysis. Suzana C. Almeida and Ivanízia S. da Silva were involved in data analysis. Suzana C. Almeida was involved in article drafting. Vivian Mara G. O. Azevedo was involved in supervision. Ingrid G. Azevedo, Ivanízia S. da Silva, Rayany C. de Souza, and Vivian Mara G. O. Azevedo critically revised the manuscript for methodological, results, and intellectual content.

## Funding

The authors received no specific funding for this work.

## Conflicts of Interest

The authors declare no conflicts of interest.

## Supporting information


**E‐File 1:** Data extraction form. **E‐Table 1:** Search strategies. **E‐Table 2:** Protocol modifications. **E‐Table 3:** Exclusions with reasons. **E‐Table 4:** Characteristics of studies ongoing. **E‐Table 5:** Characteristics of studies awaiting assessment. **E‐Table 6:** Further characteristics of included trials. **E‐Table 7:** Differences between the sedation and weaning protocols. **E‐Table 8:** Risk of bias summary review authors' judgments about each risk of bias item for each included study. **E‐Table 9:** Funding and conflict of interest. **E‐Table 10:** Sensitivity analyses by definitions of extubation failure outcome.

## Data Availability

The data that support the findings of this study are available in the Supporting Material of this article.
