# Supplementary material for: Ventilation‐Weaning Protocols in Children Admitted to Pediatric Intensive Care Units: Systematic Review of Randomized Trials
Source: Pediatr Pulmonol. 2025 Dec 22;60(12):e71436. doi: 10.1002/ppul.71436 (PMC12719932; doi:10.1002/ppul.71436)
Supplement: Supplementary file 1 — E‐File 1: Data extraction form. E‐Table 1: Search strategies. E‐Table 2: Protocol modifications. E‐Table 3: Exclusions with reasons. E‐Table 4: Characteristics of studies ongoing. E‐Table 5: Characteristics of studies awaiting assessment. E‐Table 6: Further characteristics of included trials. E‐Table 7: Differences between the sedation and weaning protocols. E‐Table 8: Risk of bias summary review authors' judgments about each risk of bias item for each included study. E‐Table 9: Funding and conflict of interest. E‐Table 10: Sensitivity analyses by definitions of extubation failure outcome. [file PPUL-60-0-s001.docx]

**SUPPLEMENTARY MATERIAL**

**Title**

Ventilation-Weaning Protocols in Children Admitted to Pediatric Intensive Care Units: Systematic Review of Randomized Trials

**Authors**

Suzana.C. Almeida M.Sc, Rayany.C. de Souza M.Sc, Ingrid.G. Azevedo Ph.D, Ivanízia.S. da Silva Ph.D, Vivian.M.G.O. Azevedo Ph.D^*^

**Table of Contents**

[E-Table 1 Search strategies 2](#_Toc214357130)

[E-File 1 Data extraction form 3](#_Toc214357131)

[E-Table 2 Protocol modifications 6](#_Toc214357132)

[E-Table 3 Exclusions with reasons 7](#_Toc214357133)

[E-Table 4 Characteristics of studies ongoing 8](#_Toc214357134)

[E-Table 5 Characteristics of studies awaiting assessment 9](#_Toc214357135)

[E-Table 6 Further characteristics of included trials 10](#_Toc214357136)

[E-Table 7 Differences between the sedation and weaning protocols 12](#_Toc214357137)

[E-Table 8 Risk of bias summary review authors' judgments about each risk of bias item for each included study 15](#_Toc214357138)

[E-Table 9 Funding and conflict of interest 22](#_Toc214357139)

[E-Table 10 Sensitivity analyses by definitions of extubation failure outcome 23](#_Toc214357140)

*Corresponding author: viviangazevedo1@gmail.com.

# E-Table 1 Search strategies

| **Database** | **Search strategy** |
| --- | --- |
| PUBMED | ("ventilator weaning"[mh] OR respirator weaning[mh] OR weaning, respirator[mh] OR mechanical ventilat* weaning OR "airway extubation"[mh] OR airway extubations[mh] OR extubat*[tiab] OR tracheal extubation[tiab] OR endotracheal extubation[mh]) AND (intensive care units, pediatric[mh] OR intensive care unit, pediatric[mh] OR pediatric intensive care units[mh] OR pediatric intensive care unit OR pediatric OR infant OR child, preschool OR child OR children OR adolescent) AND (clinical protocols[mh] OR clinical protocol[mh] OR treatment protocols[mh] OR treatment protocol[mh] OR weaning protocol [all fields] OR protocol* OR clinical practice guideline[mh] OR practice guideline[mh] OR "patient care management") AND (randomi?ed controlled trial [tiab] OR randomized controlled trial[pt] OR controlled clinical trial[pt] OR clinical trial[pt] OR random*[tiab] OR RCT[tiab]) |
| EMBASE | (‘ventilator weaning’/exp OR ‘respirator weaning’/exp OR ‘ventilation weaning’ OR ‘mechanical ventilat$ weaning’ OR ‘wean off mechanical ventilation’ OR extubat$ OR ‘tracheal extubation’/exp) AND (‘pediatric intensive care unit’/exp OR ‘intensive care units, pediatric’ OR ‘intensive care units, paediatric’ OR pediatric OR paediatric OR infant OR child OR children OR adolescent) AND (‘Clinical protocol’/exp OR ‘clinical protocols’ OR ‘weaning protocol’/exp OR protocol$ OR ‘protocol$ adj5 weaning’ OR ‘practice guideline’/exp OR ‘patient care management’/exp) AND (randomized controlled trial/exp OR controlled clinical trial:ab,ti OR randomized:ab,ti OR random$:ab,ti) |
| CINAHL | (TX ventilator weaning OR TX respirator weaning OR TX weaning, respirator OR TX mechanical ventilat* weaning OR TX airway extubation OR TX airway extubations OR TX extubat* OR TX tracheal extubation OR TX endotracheal extubation OR mechanical ventilat* weaning) AND (TX intensive care units, pediatric OR TX intensive care unit, pediatric OR TX pediatric intensive care units OR pediatric intensive care unit OR pediatric OR infant OR child, preschool OR child OR children OR adolescent) AND (TX clinical protocols OR TX clinical protocol OR TX treatment protocols OR TX treatment protocol OR TX weaning protocol OR protocol* OR MH clinical practice guideline OR MH practice guideline OR "patient care management") AND (PT randomized controlled trial OR PT controlled clinical trial OR PT clinical trial OR AB random*) |
| WEB OF SCIENCE | TOPIC=(ventilator weaning OR respirator weaning OR weaning, respirator OR airway extubation OR airway extubations OR extubat* OR tracheal extubation OR endotracheal extubation OR mechanical ventilat* weaning) AND  ALL FIELDS= (intensive care units, pediatric OR intensive care unit, pediatric OR pediatric intensive care units OR pediatric intensive care unit OR pediatric OR infant OR child, preschool OR child OR children OR adolescent) AND  TOPIC= (clinical protocols OR clinical protocol OR treatment protocols OR treatment protocol OR weaning protocol OR protocol* OR clinical practice guideline OR practice guideline OR "patient care management") AND TOPIC= (randomized controlled trial OR controlled clinical trial OR clinical trial OR random* OR RCT) |
| COCHRANE | ventilator weaning OR respirator weaning OR weaning, respirator OR airway extubation OR airway extubations OR extubat* OR tracheal extubation OR endotracheal extubation OR mechanical ventilat* weaning in Title Abstract Keyword AND intensive care units, pediatric OR intensive care unit, pediatric OR pediatric intensive care units OR pediatric intensive care unit OR pediatric OR infant OR child, preschool OR child OR children OR adolescent in Title Abstract Keyword AND clinical protocols OR clinical protocol OR treatment protocols OR treatment protocol OR weaning protocol OR protocol* OR clinical practice guideline OR practice guideline OR "patient care management" in Title Abstract Keyword AND randomized controlled trial OR controlled clinical trial OR clinical trial OR random* OR RCT in Publication Type |

E-File 1 Data extraction form
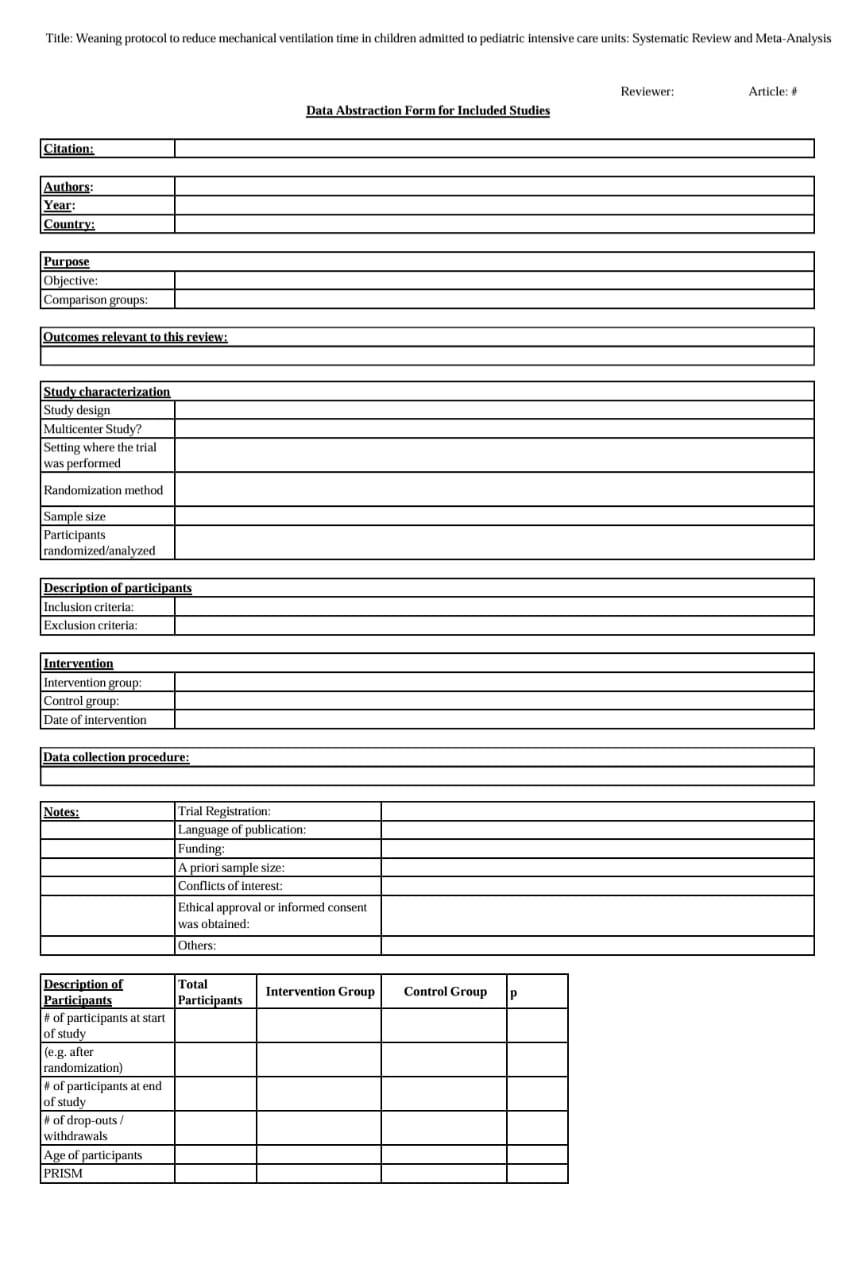


**
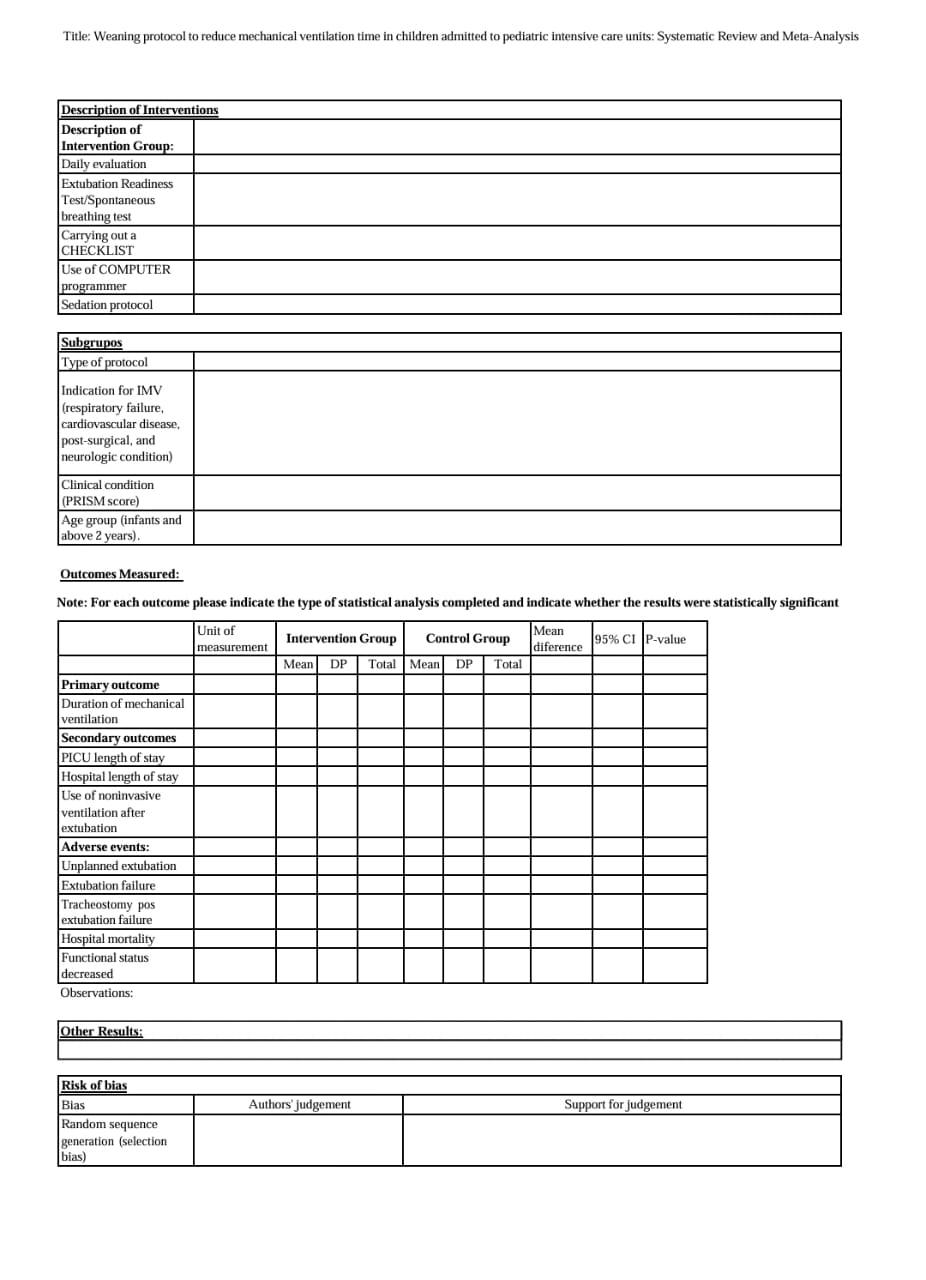
**

**
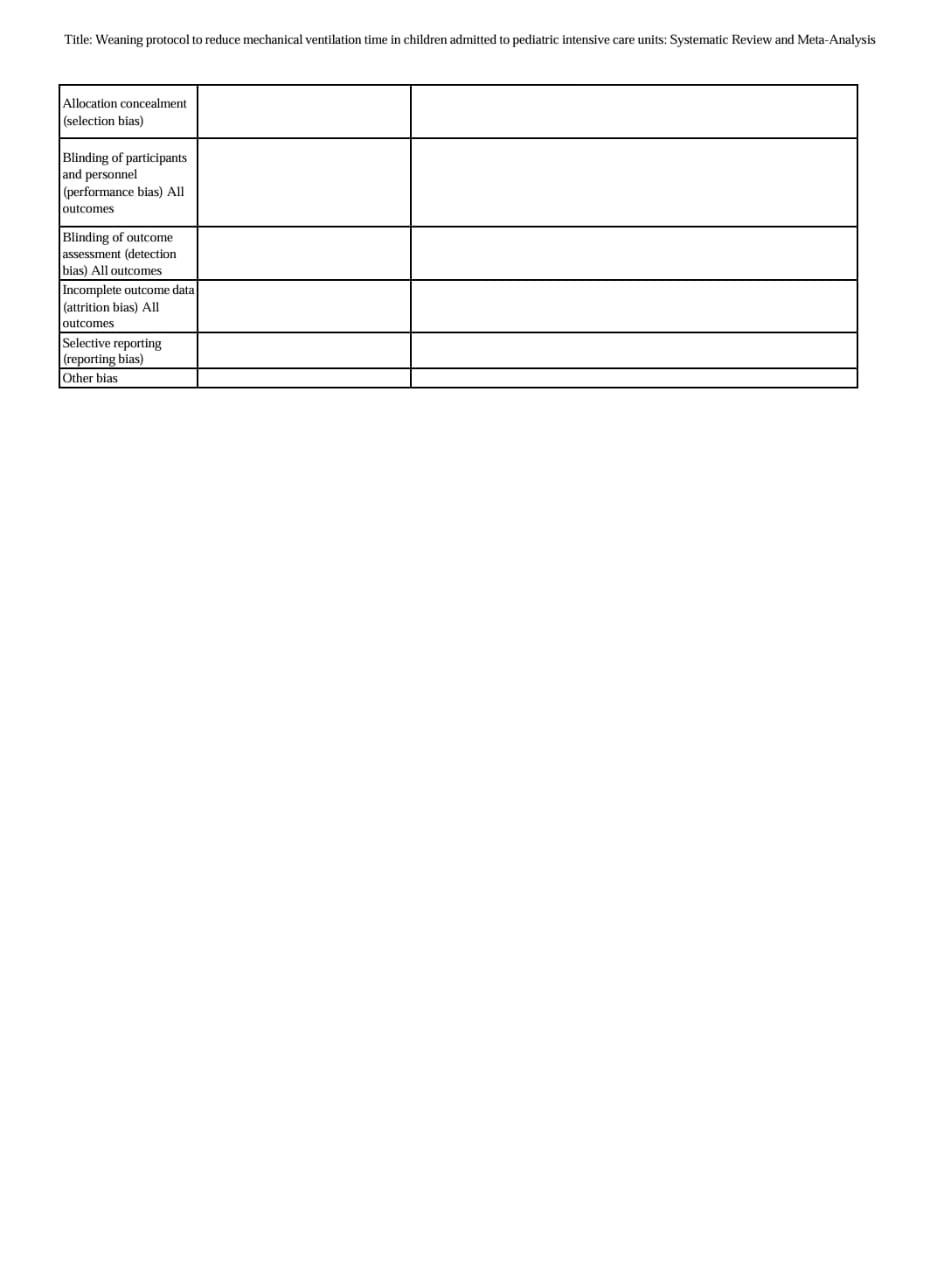
**

# E-Table 2 Protocol modifications

| **Nature of the deviation** | **Description of the deviation** | **Reason for the deviation** |
| --- | --- | --- |
| Unit of measurement for the duration of mechanical ventilation | The original protocol described the mechanical ventilation time as being measured in hours. | Some studies were analyzed in days and others in hours. After discussion with the research team, we decided to present the data in days, because the conversion would provide more reliable data. |
| Subgroup analysis | The original protocol described these subgroups analysis: type of protocol; clinical condition; and age group (Infants and above 2 years) | Subgroup analysis was not possible due to methodological and intervention differences among the studies, so the data were presented separately |
| Sensitivity analysis | The protocol planned to perform sensitivity analysis by removing studies with some concerns or high risk of bias. | Sensitivity analysis by risk of bias was not possible due to methodological and intervention differences among the studies, so the data were presented separately. |
| Sensitivity analysis | The protocol did not specify additional sensitivity analyses | Two studies defined extubation failure differently from the definition adopted in this review; therefore, we conducted a sensitivity analysis to assess whether these variations influenced the overall outcome. |

# E-Table 3 Exclusions with reasons

| **Study** | **Format** | **DOI/reference** | **Reason for exclusion** |
| --- | --- | --- | --- |
| Farias et al, 2001 | Full-text | 10.1007/s001340101035 | Comparison of two spontaneous breathing test methods. Did not compare weaning protocol with usual care |
| Hotz et al, 2020 | Full-text | 10.1097/PCC.0000000000002556 | Not randomized |
| Vallabhaneni et al, 2023 | Full-text | 10.1007/s12098-023-04959-1. | The intervention was only a sedation protocol |

# E-Table 4 Characteristics of studies ongoing

| **Khemani et al, 2020** | |
| --- | --- |
| DOI/reference | 10.1016/j.cct.2019.105893. |
| Clinical Trials ID | NCT03266016 |
| Methods | Single-center, randomized controlled trial |
| Participants | Setting: Children’s Hospital Los Angeles Inclusion criteria: Children > 1 month (at least 44 weeks corrected gestational age) and ≤ 18 years of age requering invasive mechanical ventilation for pulmonary parenchymal disease for at least 48 hours. Exclusion criteria: Contraindications to an esophageal catheter; contraindications to use of respiratory inductance plethysmography bands; conditions precluding diaphragm ultrasound measurement; conditions precluding conventional methods of weaning (i.e., status asthmaticus, severe lower airway obstruction, critical airway, intracranial hypertension, extra corporeal life support, limitation of care, severe chronic respiratory failure, spinal cord injury above lumbar region, cyanotic heart disease); primary attending physician refusal. Participant numbers: target 300 children (150 children per arm). |
| Interventions | Protocol: Patients will be managed in a synchronized intermittent mandatory ventilation mode with pressure control plus Pressure Support with a computerized decision support tool recommending changes to ventilator settings every 4 hours or with a new blood gas Control: Ventilator management will be per usual care until the patient meets weaning criteria and passes the oxygenation test. |
| Outcomes | • Duration of weaning from invasive mechanical ventilation; • 28-day ventilator free days; • Extubation failure. |
| Notes | The trial has completed in March . It is undergoing peer review currently (reported by author). |
| **Pudjiadi, 2021^*^** |  |
| Clinical Trials ID | NCT04788589 |
| Methods | Single-blinded, randomized controlled trial |
| Participants | Setting: Cipto Mangunkusumo Hospital, Indonesia. Inclusion criteria: Children (1 month to 17 years) who need mechanical ventilation for more than 12 hours. Exclusion criteria: Pediatric Intensive Care Unit admission due to post cardiac and respiratory arrest. Participant numbers: not informed |
| Interventions | Protocol: sedation and ventilator weaning protocol (sedation control and spontaneous breathing test with Continuous Positive Airway Pressure) Control: Sedation and ventilator weaning according to attending physicians |
| Outcomes | • Pain and sedation score • Ventilator days • Self extubation • Reintubation • PICU Length of stay |
| Notes | Unable to contact the author. Protocol from clinicaltrials.gov |
| * Unpublished |  |

# E-Table 5 Characteristics of studies awaiting assessment

| **Randolph et al, 2002** | |
| --- | --- |
| DOI/reference | 10.1001/jama.288.20.2561 |
| Methods | Multi-centre, randomized controlled trial |
| Participants | Setting: Pediatric intensive care units of 10 children’s hospitals across North America Inclusion criteria: Children (aged <18 years) in pediatric intensiva care unit required ventilator support for more than 24 hours. Exclusion criteria: Corrected gestational age <38 weeks, diaphragmatic hernia or paralysis, ventilator use prior to admission, cyanotic congenital heart disease with unrepaired or palliated right-to-left intracardiac shunt, history of single ventricle defect, significantly diminished lung capacity, decreased lung vascularity, anatomical obstruction of lower airways, primary pulmonary hypertension or anticipated need for nitric oxide after extubation, previous bone marrow or lung transplant, spinal cord injury above the lumbar region, tracheal or upper airway obstructive conditions, status asthmaticus in children 2 years or older, and progressive neuromuscular weakness; currently enrolled in another trial; decision to withdraw or limit life support. Participant numbers: 182 randomized / 179 analyzed |
| Interventions | Protocol: A protocolized reduction in ventilatory parameters was carried out and then Extubation Readiness Test. Ventilator management in two groups: the manual adjustment of pressure support ventilation by clinicians and continuous automated adjustment of pressure support by the ventilator. Control: Weaning was at the discretion of the physician and no management recommendations were made (No protocol). |
| Outcomes | • Duration of weaning time (from randomization to successful extubation); • Extubation failure (any invasive or noninvasive ventilator support within 48 hours of extubation). |
| Notes | The sample included neonates. We await further communication regarding the ability to separate neonatal data from children's data. |
| **Schultz et al, 2001** | |
| DOI/reference | Not found |
| Methods | Prospective-randomized. |
| Participants | Setting: Pediatric and cardiac intensive care units in a 307-bed tertiary referral hospital for children. Inclusion criteria: Children required ventilator support. (Not well described in the abstract) Exclusion criteria: Not described in the abstract. Participant numbers: 223 patients |
| Interventions | Protocol: Weaning according to a predetermined algorithm. Control: Weaning according to individual physician order for reduction ventilator parameters |
| Outcomes | • Weaning time, pre-weaning time, and extubation time; • Incidence of reintubation; • Subglottic stenosis, tracheitis and pneumonia. |
| Notes | It was not possible to access the full article. We are awaiting further communication with the author. |

# E-Table 6 Further characteristics of included trials

| **Study** | **N Protocol** | **N Control** | **Age Protocol (months)** | **Age Control (months)** | **p value** | **Mortality Risk Score- Protocol*** | **Mortality Risk Score- Control*** | **p value** | **Indication of intubation** | **Protocol N(%)** | **Control N(%)** | **p value** |
| --- | --- | --- | --- | --- | --- | --- | --- | --- | --- | --- | --- | --- |
| Blackwood et al, 2021 | 4688 | 4155 | 7 (1-45) | 9 (1-47) | _ | 0.02 (0.01-0.05)^e^ | 0.02 (0.01-0.05)^e^ | _ | Did not specify the reason for intubation, reporting only the primary diagnosis | 30% for respiratory primary diagnostic | 31% for respiratory primary diagnostic | _ |
| Curley et al, 2015^a^ | 1170 | 1179 | 17.4 (3.6- 87.6) | 34.8 (8.4-114.6) | < 0.01^g^ | 6 (3-11)^c^ | 9 (5-14)^c^ | < 0.01^g^ | Only respiratory disease | _ | _ | _ |
| Foronda et al, 2011 | 134 | 126 | 13.92 (4.44-42.12) | 8.52 (3-26.04 | 0.07^g^ | 0.04 (0.02-0.10)^d^ 0.09 (0.03; 0.19)^f^ | 0.05 (0.03; 0.13)^d^ 0.08 (0.036; 0.18)^f^ | 0.42^g^  0.09^g^ | Congestive heart failure Wheezing Septic shock Coma Pneumonia  Bronchiolitis  Acute respiratory distress syndrome | 3 (2.2%)  34 (25.4%)  26 (19.4%) 19 (14.2%) 55 (41.0%) 28 (20.9%) 7 (5.2%) | 6 (4.8%)  30 (23.8%) 18 (14.3%) 13 (10.3%)  55 (43.7%)  32 (25.4%)  6 (4.8%) | 0.27^h^ 0.77 0.27 0.34 0.67 0.39 0.86 |
| Jouvet et al, 2013 | 15 | 15 | 119 (±53)^b^ | 116 (±57)^b^ | 0.92^g^ | 5.7 ± 5.3^b,f^ | 8.6 ± 18.4^b,f^ | 0.31^g^ | Pulmonary failure  Heart failure Coma  Postoperative  Trauma  Sepsis  Other | 8 2  3  6  3  0  2 | 8 6  3  4  1  2 0 | 1.00^g^ 0.21 1.00 0.43 0.60 0.48 0.48 |
| Keivanfar et al, 2020 | 34 | 34 | 16.5(6-59) | 20(4.7-85.5) | 0.77^g^ | 3(0-9)^d^ | 3(0.75-6)^d^ | 0.82^g^ | Acute respiratory failure  Loss of consciousness | 21(61.8%) 13(38.2%) | 19(55.9%) 15(44.1%) | 0.4^h^ 0.4 |
| Kishore and Jhamb, 2021 | 38 | 38 | 11.5 (3–54) | 45 (6.5–102) | 0.02^g^ | _ | _ | _ | Acute respiratory failure (pulmonary disease) Acute respiratory failure (neurological disease) Altered mental state Glasgow Coma Scale <8 Cardiovascular disease Shock Elective ventilation (postoperatively) | 16 (40%)  5 (12.5%)  3 (7.5%)  3 (7.5%) 1 (2.5%) 12 (30%) | 14 (35%)  7 (17.5%)  7 (17.5%)  1 (2.5%) 1 (2.5%) 10 (25%) | _ |
| Maloney, 2007 | 15 | 16 | 1.4 (0.9 -13.9) | 3.2 (1.3-39.3) | 0.19^g^ | 7.0 (±3.9)^b,c^ | 5.9 (±4.8)^b,c^ | 0.51^i^ | Only respiratory disease | _ | _ | _ |

N= number of patients; Data on patients' age and mortality risk were presented as Median (IQR 25th-75th), except in the studies by Jouvet et al. and Maloney.

a Data presented already excluding neonates and tracheostomized patients

b Mean (±SD)

c- PRISM III

d- PRISM II

e- PIM 3

f- PIM 2

g- Mann-Whitney U test

h- Chi-square test

i- T-test

# E-Table 7 Differences between the sedation and weaning protocols

| **Study** | **Weaning sedation Protocol** | **Weaning ventilation Protocol** | **Readiness screenings** | **Spontaneous breathing test** | **Failure criteria in Spontaneous breathing test** |
| --- | --- | --- | --- | --- | --- |
| Blackwood et al, 2021 | Assessment of sedation levels by the bedside nurse using the COMFORT scale score (every 6 hours as a minimum time interval). | Daily readiness screenings and spontaneous breathing test | • FiO2 ≤0.45;  • SpO2 ≥95% (or as appropriate);  • PEEP level ≤8cm H2O;  • PIP ≤22 cmH2O;  • Cough present. | •Continuous positive airway pressure mode;  • PEEP of 5 cmH_2_O, or PS of at least 5 cmH_2_O in addition to PEEP; • For a maximum of 2 hours. | • 20% increase in heart or respiratory rate. • Signs of increased work of breathing; • SpO_2_ < 92% or significant sustained increase in FiO_2_ requirement. |
| Curley et al, 2015 | A goal-directed algorithm was used to guide sedation therapy, which included daily discussions of the patient's illness trajectory, setting a target SBS score, and adjusting sedatives based on the illness phase at least every 8 hours. | Extubation readiness test | • Spontaneous breathing; • Oxygenation Index < 6; • Decrease and/or plateau in ventilator support over the previous 12 hrs. | • FiO2 of 0.5;  • PEEP of 5cmH2O;  • PS: 10cmH2O if ETT 3-3.5mm; 8cmH2O if ETT 4-4.5mm; 6cmH2O if ETT ≥5mm;  • For 2 hrs | • Spo_2_ <95% • Exhaled tidal volume <5ml/kg • Respiratory rate outside the normal for the age group. |
| Foronda et al, 2011 | _ | Daily readiness screenings and spontaneous breathing test | • FIO2 ≤50%;  • PEEP ≤8 cm H2O;  • PIP ≤25 cm H2O;  • Absence of new infiltrates on the chest radiograph;  • Presence of respiratory drive;  • No continuous sedation;  • No use of NMB in the last 24 hrs;  • Correction of electrolyte changes;  • Hemodynamic stability  • Hemoglobin 8g/dL. | • PS mode • PEEP of 5 cm H_2_O; • PS of 10 cmH_2_O,  • FIO_2_ that was used before the test; • For 2 hrs. | • Respiratory or heart rate increase 20% above the initial values;  • Signs of increased respiratory work; • Changes in the level of consciousness; • Blood pressure <fifth percentile for patient’s age; • SaO_2_ <90%, or PaCO_2_ >50 mmHg (or an increase >10 mmHg in 1 hr in patients with chronic lung disease). |
| Jouvet et al, 2013 | _ | Decrease ventilatory support with computerized pediatric ventilator weaning protocol: SmartCare/PSTM | Carried out in both groups:  • Ability to breath spontaneously;  • No vasopressor or inotrope;  • FiO2 ≤60% with SpO2 ≥95%;  • PEEP ≤8cmH2O;  • Plateau pressure ≤25cmH2O;  • EET leak ≤20%. | Carried out in both groups:  • PS mode;  • ±5 cmH_2_O of the pre-inclusion plateau pressure. | Not mentioned |
| Keivanfar et al, 2020 | _ | Daily assessment of arterial blood gas test to gradually reduce ventilatory parameters; evaluation of readiness criteria, and spontaneous breathing test. | • Recovery of causes for intubation;  • Temperature <38.5 oC;  • Hemoglobin ≥8;  • Gag or cough reflexes;  • Acceptable level of consciousness;  • Correction of electrolytes;  • Absence of new findings in the chest X-ray;  • No ventilator setting increase needed in the past 24 hrs;  • Discontinuation of sedation and analgesics in the previous 6 hrs;  • No need for vasoactive agents;  • SpO2>94%, despite FiO2<40, PIP<20cmH2O and PEEP<5cmH2O | • PS mode;  • PEEP≤5 cmH2O;  • FiO2≤0.4.;  • PS: 10cmH2O if ETT 3-3.5mm, 8cmH2O if ETT 4-4.5mm, 6cmH2O if ETT≥5mm | • Respiratory or heart rate outside the normal for the age group; • Respiratory distress; • Exhaled tilde volume <5 ml/kg; • SatO_2_<90%; • Hypotension based on PALS guidelines or an over 20% increase in blood pressure; • Impaired consciousness associated with hypoventilation; • PCO_2_>55 mmHg or an increase in PCO_2_ by at least 10mmHg in patients with chronic pulmonary disease. |
| Kishore and Jhamb, 2021 | _ | Daily readiness screenings and Spontaneous breathing trial (pressure support ventilation and T-piece test)*. | Carried out in both groups:  • Hemodynamically stable;  • Basic pathology improved/ resolved;  • Spontaneous breathing efforts;  • Gag or cough reflex on suctioning;  • Acceptable level of consciousness;  • Ph 7.32 to 7.47 on recent blood-gas analysis;  • PEEP ≤5 cmH2O;  • FiO2 ≤0.5;  • PIP ≤25 cmH2O;  • Absence of new infiltrates on the chest radiograph;  • No clinical need to increase ventilator support in the last 12 hrs;  • No planned operative procedure in the next 48 hrs;  • Not on paralysis in the past 24 hrs;  • Minimal sedation. | • PS mode  • PS: 10cmH2O if EET 3-3.5mm, 8cmH2O if ETT 4-4.5mm, and 6cmH2O if ETT ≥5mm;  • For 2 hrs.  • After the PS trial, a T-piece trial was performed (with oxygen if required) for 2 hrs. | • Respiratory rate outside the normal for the age group;  • Increase in heart rate >20% over baseline;  • Increase in work of breathing;  • Tidal volume ≤5 mL/kg;  • Blood pressure falls to <5th centile for age;  • Spo2 decrease ≤92%;  • ETCO2 rises >10 mmHg/hour. |
| Maloney 2007 | _ | Decrease ventilatory support and weaning trial with a computerized ventilator weaning protocol: Java platform and Blaze Advisor rules engine. | • Tidal volume < 9 ml/kg; • PEEP < 6 cmH_2_0; • FiO2 < 0.4; • mechanical respiratory rate appropriate for ETT size (ETT < 3.5 mm: 12 breaths/min, ETT 3.5- 4.0 mm: 10 breaths/min, and ETT > 4.0 mm: 8 breaths/min) with a normal patient respiratory rate; • PS, when used, < 8 cmH_2_O. | _ | _ |

*Daily readiness screenings and Spontaneous breathing test with T-piece test were performed in the intervention and control group

Legend: FIO_2_: Fraction of inspired oxygen; SpO_2_: Oxygen saturation as measured by pulse oximetry; PEEP: Positive end-expiratory pressure; PIP: Peak inspiratory pressure; PS: pressure support; SBS: State Behavioral Scale; ETT: endotracheal tube; NMB: neuromuscular blockers; SaO_2_: Arterial blood oxygen saturation; PaCO_2_: partial pressure of arterial carbon dioxide; ETCO_2_: end-tidal carbon dioxide.

# E-Table 8 Risk of bias summary review authors' judgments about each risk of bias item for each included study

|  |  | Blackwood et al, 2021 | Curley et al, 2015 | Foronda et al, 2011 | Jouvet et al, 2013 | Keivanfar et al, 2020 | Kishore and Jhamb, 2021 | Maloney, 2007 |
| --- | --- | --- | --- | --- | --- | --- | --- | --- |
| **ROB due to Randomization Process** | 1a.1 Was the allocation sequence random? | Y – computer-generated algorithm | Y – computer-generated random numbers | Y – opaque plastic bags containing varying numbers | Y- Allocated randomly by sealed envelopes (author's response). | Y – block randomization with a block size of two. | Y- computer-generated randomization list (block of eight) | Y-computer-generated randomization in blocks of ten (author response). |
|  | 1a.2 Was the allocation sequence concealed until participants were enrolled and assigned to interventions? | Y –Allocation and details of the intervention were concealed until the point of randomization. (protocol) | Y- Random assignment was conducted by the Data Coordinating Center team (protocol) | Y- Sealed envelopes prepared by staff who were not involved with patient care. | Y- The research assistant took the envelope corresponding to the patient number (author's response). | NI- no reference to concealment | Y-Sequentially arranged sealed opaque envelopes, prepared by a person who was not involved in the study | NI- no reference to concealment |
|  | 1a.3 Did baseline differences between intervention groups suggest a problem with the randomization process? | N – no apparent imbalances | PN- Baseline characteristics were similar between groups, except for age, and PRISM III score. Imbalances compatible with chance | N – no apparent imbalances | N – no apparent imbalances | PN-Baseline characteristics were similar between groups, except for the cause of admission (post-operative). Imbalance compatible with chance | PN- Baseline characteristics were similar between groups, except for age, and weight. Imbalances compatible with chance | N – no apparent imbalances |
|  | Judgement | LOW RISK | LOW RISK | LOW RISK | LOW RISK | SOME CONCERNS | LOW RISK | SOME CONCERNS |
| **ROB in Timing of identification or recruitment of participants*** | 1b.1 Were all the individual participants identified and recruited (if appropriate) before randomization of clusters? | N-All clusters was recruite prior to starting the trial to enable all units to begin baseline data collection at the same time point (from protocol) | NI- But they used a cluster randomization design, not an individual recruitment | Does not apply | Does not apply | Does not apply | Does not apply | Does not apply |
|  | 1b.2 Is it likely that selection of individual participants was affected by knowledge of the intervention assigned to the cluster? | N-The allocation and intervention details were concealed until the randomization process (from protocol). | N- The cluster only knew which group it went to after randomization. All eligible participants from the unit were included. | Does not apply | Does not apply | Does not apply | Does not apply | Does not apply |
|  | 1b.3 Were there baseline imbalances that suggest differential identification or recruitment of individual participants between intervention groups? | N- no apparent imbalances | PN-Baseline characteristics were similar between groups, except for age, PRISM III score, and primary diagnosis. Imbalances compatible with chance | Does not apply | Does not apply | Does not apply | Does not apply | Does not apply |
|  | Judgement | LOW RISK | LOW RISK | _ | _ | _ | _ | _ |
| **ROB due to deviations from intended interventions** | 2.1a Were participants aware that they were in a trial? * | PN-Patients are unaware of the intervention because they are sedated | PN-Patients are unaware of the intervention because they are sedated | Does not apply | Does not apply | Does not apply | Does not apply | Does not apply |
|  | 2.1b Were participants aware of their assigned intervention during the trial? | N/A | N/A | PN-Patients are unaware of the intervention because they are sedated | PN-Patients are unaware of the intervention because they are sedated | N- Patients were not aware of respiratory care | PN-Patients are unaware of the intervention because they are sedated | PN-Patients are unaware of the intervention because they are sedated |
|  | 2.2 Were carers and people delivering the interventions aware of participants' assigned intervention during the trial? | Y-Non-blind study | Y-Non-blind study | Y- Non-blind study | Y- Non-blind study | Y- Single-blind study | Y- Non-blind study. | Y- Non-blind study. |
|  | 2.3 Were there deviations from the intended intervention that arose because of the trial context? | PN- The reasons for non-adherence to protocol components were described in supplementary tables, and do not appear to be related to the trial context. | PN- It does not mention the reasons why there was no adherence to the protocol in some cases, but it does not seem to be related to the trial context. | PN- The author describes in the article all patients who had deviations from the intervention, not characterized as due to the context of the trial. | PN- The author describes the reasons for the delay when the computer suggested extubation, and do not appear to be related to the trial context. | NI- The author does not inform whether there were deviations from the intervention. | PN- The author describes all patients who had deviations from the intervention. They are not related to the trial context | PN- The author describes why there was a deviation from the intended interventions. They are not related to the trial context |
|  | 2.4 Were these deviations likely to have affected the outcome? | N/A | N/A | N/A | N/A | N/A | N/A | N/A |
|  | 2.5 Were these deviations from intended intervention balanced between groups? | N/A | N/A | N/A | N/A | N/A | N/A | N/A |
|  | 2.6 Was an appropriate analysis used to estimate the effect of assignment to intervention? | Y - ITT | Y- ITT | Y - ITT | Y – ITT | Y- ITT | Y- ITT | Y- ITT |
|  | 2.7 Was there potential for a substantial impact (on the result) of the failure to analyse participants in the group to which they were randomized? | N/A | N/A | N/A | N/A | N/A | N/A | N/A |
|  | Judgement | LOW RISK | LOW RISK | LOW RISK | LOW RISK | SOME CONCERNS | LOW RISK | LOW RISK |
| **ROB due to missing outcome data** | 3.1a Were data for this outcome available for all clusters that recruited participants?* | Y | Y | Does not apply | Does not apply | Does not apply | Does not apply | Does not apply |
|  | 3.1b Were data for this outcome available for all, or nearly all, participants randomized? | Y-Recruitment and attrition were reported | Y-Recruitment and attrition were reported | Y-Recruitment and attrition were reported | Y- Results were presented for all patients randomized into each group | Y- Results were presented for all patients randomized into each group | Y-Recruitment and attrition were reported | Y-Recruitment and attrition were reported |
|  | 3.2 Is there evidence that the result was not biased by missing outcome data? | N/A | N/A | N/A | N/A | N/A | N/A | N/A |
|  | 3.3 Could missingness in the outcome depend on its true value? | N/A | N/A | N/A | N/A | N/A | N/A | N/A |
|  | 3.4 Is it likely that missingness in the outcome depended on its true value? | N/A | N/A | N/A | N/A | N/A | N/A | N/A |
|  | Judgement | LOW RISK | LOW RISK | LOW RISK | LOW RISK | LOW RISK | LOW RISK | LOW RISK |
| **ROB in measurement of outcome** | 4.1 Was the method of measuring the outcome inappropriate? | N- The method of measuring the outcome was appropriate | N- The method of measuring the outcome was appropriate | N- The method of measuring the outcome was appropriate | N- The method of measuring the outcome was appropriate | N- The method of measuring the outcome was appropriate | N- The method of measuring the outcome was appropriate | N- The method of measuring the outcome was appropriate |
|  | 4.2 Could measurement or ascertainment of the outcome have differed between intervention groups? | N- The data collection involves the same measurement methods in two groups | N- The data collection involves the same measurement methods in two groups | N- The data collection involves the same measurement methods in two groups | N- The data collection involves the same measurement methods in two groups | N- The data collection involves the same measurement methods in two groups | N- The data collection involves the same measurement methods in two groups | N- The data collection involves the same measurement methods in two groups |
|  | 4.3a Were outcome assessors aware that a trial was taking place?* | Y- Unblinded | Y- Unblinded | Does not apply | Does not apply | Does not apply | Does not apply | Does not apply |
|  | 4.3b Were outcome assessors aware of the intervention received by study participants? | Y- Unblinded | Y- Unblinded | N- The assessments were made by a person who did not participate in the intervention | N- Those involved in outcome assessment were blinded to the allocated intervention (author's response) | Y- Single-blinding | Y- Non-blind study. | N- The data safety monitoring board was blinded to allocation when they performed the analysis (author response) |
|  | 4.4 Could assessment of the outcome have been influenced by knowledge of intervention received? | PN- The intervention occurred after the control assessment, with protocol adherence required for decision-making. | PN- The intervention cluster did not know what was being done in the control cluster, and vice versa | N/A | N/A | PY- Possibly due to the nature of the study (involves the researcher's judgment in performing extubation) | PY- Possibly due to the nature of the study (involves the researcher's judgment in performing extubation) | N/A |
|  | 4.5 Is it likely that assessment of the outcome was influenced by knowledge of intervention received? | N/A | N/A | N/A | N/A | PN- one person delivered the intervention, and the team delivered usual care | PN- Daily screening, randomization, and SBT in the intervention group was performed by a designated resident, while extubation were done by the managing team | N/A |
|  | Judgement | LOW RISK | LOW RISK | LOW RISK | LOW RISK | SOME CONCERNS | SOME CONCERNS | LOW RISK |
| ROB in reported results | 5.1 Were the data that produced this result analysed in accordance with a pre-specified analysis plan? | Y- Protocol was registered as ISRCTN16998143 and outcomes were reported. | Y-Protocol was registered as NCT00814099, and outcomes were reported | Y- Protocol was registered as ISRCTN37806223 and outcomes were reported | Y- Protocol was registered as NCT00678912 and outcomes were reported | Y- Protocol was registered as IRCT20190219042766N1 and outcomes were reported | PY- Clinical trial registration number (CTRI/2018/04/013270) does not match the article. But the article's analyses align with the methods section. | PY- No protocol was provided, but outcomes relevant to trials are reported in the thesis |
|  | 5.2 Is the numerical result being assessed likely to have been selected, on the basis of the results, from multiple eligible outcome measurements (e.g. scales, definitions, time points) within the outcome domain? | N- There is only one possibly way this outcome was measured | N - There is only one possibly way this outcome was measured | N- There is only one possibly way this outcome was measured | N - There is only one possibly way this outcome was measured | N- There is only one possibly way this outcome was measured | N- There is only one possibly way this outcome was measured | N- There is only one possibly way this outcome was measured |
|  | 5.3 Is the numerical result being assessed likely to have been selected, on the basis of the results, from multiple eligible analyses of the data? | N – Followed the plan | N – Followed the plan | N- Followed the plan | N- Followed the plan | N- Followed the plan | N – Followed the plan | N- Followed the plan |
|  | Judgement | LOW RISK | LOW RISK | LOW RISK | LOW RISK | LOW RISK | LOW RISK | LOW RISK |
| Overall ROB |  | LOW RISK | LOW RISK | LOW RISK | LOW RISK | SOME CONCERNS | SOME CONCERNS | SOME CONCERNS |
| *Only for clusters | |  |  |  |  |  |  |  |

# E-Table 9 Funding and conflict of interest

| **Study** | **Fundyng** | **Conflict of Interest** |
| --- | --- | --- |
| Blackwood et al, 2021 | Trial commissioned and funded by the National Institute for Health Research and supported by the Paediatric Critical Care Society Study Group. The Queen’s University Belfast took legal responsibility for all aspects of the research but did not provide specific funding. Role of the Funder/Sponsor: The National Institute for Health Research Health approved the design of the study and monitored the conduct of the study. It played no direct role in the design, data collection, management, analysis, and interpretation of the data; preparation, review, or approval of the manuscript; or decision to submit the manuscript for publication | Dr Clarke reported being the director of the Northern Ireland Clinical Trials Unit. No other disclosures were reported. |
| Curley et al, 2015 | The study was supported by grants from the National Heart, Lung, and Blood Institute (U01 HL086622 to Dr. Curley and U01 HL086649 to Dr. Wypij). Supported by grants from the National Heart, Lung, and Blood Institute and the National Institute of Nursing Research, National Institutes of Health (U01 HL086622 to Dr. Curley and U01 HL086649 to Dr. Wypij). | Not declared |
| Foronda et al, 2011 | Not informed | The authors have not disclosed any potential conflicts of interest. |
| Jouvet et al, 2013 | The ‘Réseau en Santé Respiratoire du FRSQ’ (Québec) supported this pilot study financially and Drager Medical (Lubeck, Germany) provided the ventilator (Evita XLTM) equipped with the Expert Weaning System: SmartCare/PSTM. | Drager Medical was not involved in the design of the study; in the collection, analysis, or interpretation of the data; in the preparation of the manuscript, or in the decision to submit the manuscript for publication. |
| Keivanfar et al, 2020 | Not informed | None |
| Kishore and Jhamb, 2021 | None | None |
| Maloney, 2007 | Funds were not available to compensate the Consensus Committee members for night and weekend validations. Consensus Committee members agreed to evaluate the protocol "for free" from 7 AM to 8 PM during weekdays (pag. 112). Respiratory care administration provided financial resources to purchase the pagers used to alert the RTs that an instruction was available (pag. 122). | Not declared |

# E-Table 10 Sensitivity analyses by definitions of extubation failure outcome

| **Type of protocol** | **Definitions of extubation failure** | **Effect estimate** |
| --- | --- | --- |
|  | All definitions | RR= 0.95 (95% CI, 0.85, 1.05) |
| Weaning sedation plus ventilation protocol | Only Reintubation within 48 hours | RR= 0.95 (95% CI, 0.85, 1.07) |
|  | Only Reintubation within 36 hours | RR= 0.94 (95% CI, 0.72, 1.23) |
|  | All definitions | RR= 1.36 (95% CI, 0.33, 5.69) |
| Automated-driven weaning protocol | Reintubation within 48 hours | RR= 2.00 (95% CI, 0.20, 19.78) |
|  | Reintubation within 24 hours | RR= 1.07 (95% CI, 0.17, 6.64) |
|  |  |  |
